# The Mitochondrial Genome of *Eleusine indica* and Characterization of Gene Content within Poaceae

**DOI:** 10.1093/gbe/evz229

**Published:** 2019-10-23

**Authors:** Nathan D Hall, Hui Zhang, Jeffrey P Mower, Joseph Scott McElroy, Leslie R Goertzen

**Affiliations:** 1 Department of Biological Sciences, Auburn University; 2 Department of Crop, Soil and Environmental Sciences, Auburn University; 3 Department of Agronomy and Horticulture, University of Nebraska-Lincoln

**Keywords:** *Eleusine*, mitochondrial genome, rpl2, Poaceae, gene loss

## Abstract

Plant mitochondrial (mt) genome assembly provides baseline data on size, structure, and gene content, but resolving the sequence of these large and complex organelle genomes remains challenging due to fragmentation, frequent recombination, and transfers of DNA from neighboring plastids. The mt genome for *Eleusine indica* (Poaceae: goosegrass) is comprehensibly analyzed here, providing key reference data for an economically significant invasive species that is also the maternal parent of the allotetraploid crop Finger millet (*Eleusine coracana*). The assembled *E. indica* genome contains 33 protein coding genes, 6 rRNA subunits, 24 tRNA, 8 large repetitive regions 15 kb of transposable elements across a total of 520,691 bp. Evidence of RNA editing and loss of *rpl2*, *rpl5, rps14*, *rps11*, *sdh4*, and *sdh3* genes is evaluated in the context of an updated survey of mt genomic gene content across the grasses through an analysis of publicly available data. Hypothesized patterns of Poaceae mt gene loss are examined in a phylogenetic context to clarify timing, showing that *rpl2* was transferred to the nucleus from the mitochondrion prior to the origin of the PACMAD clade.

## Introduction

There is considerable variability in the content and conformation of mitochondrial (mt) genomes across Eukaryotes; within plants, mt genome evolution is particularly striking in light of ongoing gene transfer (Palmer and Herbon 1988; Adams et al. 2000; Palmer et al. 2000; Adams and Palmer 2003; Smith and Keeling 2015). Because the endosymbiotic origin of mitochondria, roughly 1,000 genes have been transferred to the nucleus leaving only 3–67 genes to compose the organelle genome (Adams and Palmer 2003). This transfer has halted in metazoans owing to a shift in codon usage that occurred 600 Ma, with their mt genome content relatively fixed as a result (Boore 1999; Adams and Palmer 2003; Cantara et al. 2013). Mitochondrial translation in green plants uses the universal genetic code, allowing for the ongoing transfer and viability of mt genes in the nucleus. As such, plants offer an ideal window into the evolutionary dynamics that shape mt genomes and their content. 

Two additional differences between animal and plant mitochondria are precipitated by frequent recombination within plant mt genomes: Low rates of gene collinearity and low rates of substitution (Sloan 2013; Christensen 2013; Gualberto et al. 2014). The rate of recombination among mt genetic segments promotes gene shuffling to the point where collinearity is essentially meaningless (Iorizzo et al. 2012). Sloan et al. (2012) also demonstrate an inverse relationship between the rate of recombination and the rate of substitution. Recombination among subgenomic segments paired with stabilizing selection results in extremely low rates of substitution along with the continual rearrangement observed within plant mt (Palmer and Herbon 1988; Christensen 2014; Gualberto et al. 2014), in marked contrast to the pattern observed in animals (Boore 1999).

The transfer of mt genes to the nucleus is ongoing in plants and requires multiple steps including the fixation of any RNA edits and the acquisition of promoter sequence (Wu et al. 2017). Successful transfers eventually lead to the elimination of mt gene copies, but numerous examples have been described in a transitional state. In these cases, a species either maintains a functional nuclear and mt gene, for example, *sdh4* from *Populus* (Choi et al. 2006), *rpl5* from *Triticum aestivum* and other grasses (Sandoval et al. 2004) and *rps19* from *Bromus inermis* (Atluri et al. 2015), or they retain a mt pseudogene as observed for *rps14* from *Arabidopsis* (Aubert et al. 1992) and select grasses (Ong and Palmer 2006). In addition to the physical incorporation of DNA into the nucleus (near a promoter and mt targeting sequence), RNA edits must also be fixed to correctly code for a protein (Brennicke et al. 1993; Adams and Palmer 2003; Bonen 2006). Flowering plant mt genomes experience striking rates of RNA editing, in the range of 300–600 edit sites, compared with average plastid mRNA at a total of 20–100 (Ichinose and Sugita 2016). RNA editing occurring on organellar transcripts generally changes the coded for amino acid by C-to-U edits (Ichinose and Sugita 2016), though there are cases of edits made to group II introns (Bonen 2008), rRNA (Schuster et al. 1991), and tRNA (Schuster et al. 1991; Fey et al. 2002). Edits are made by the editosome that comprises nuclear-encoded genes including pentatricopeptide repeat proteins (Lurin 2004) and multiple other organellar RNA-editing factors (Takenaka et al. 2014). RNA editing has been extensively reviewed (Fey et al. 2002; Takenaka et al. 2013; Ichinose and Sugita 2016) and is of interest when investigating the mt genome and the process of gene transfer (Bonen 2006; Wu et al. 2017).

A final and interesting facet of plant mt genomes is the uptake and preservation of non-mt DNA, frequently plastid in origin (Lloyd et al. 2012). *Amborella* provides an extreme example of this phenomenon containing full genomes worth of foreign DNA from co-occurring epiphytes, algae, mosses, and other angiosperms (Rice et al. 2013). The uptake of foreign DNA by mt is facilitated by the permeability transition pore complex, which shows little, if any, discrimination in the DNA fragments it will import (Koulintchenko et al. 2003). The multipartite nature and frequent recombination of the mt genome likely promotes its incorporation and preservation (Rice et al. 2013). Although, foreign DNA is typically nonfunctional (Mower et al. 2010; Wang et al. 2012), in rare cases plastid tRNA transfers to the mt do function in a transcriptional capacity (Kanno et al. 1997; Miyata et al. 1998). Plastid insertions are of particular concern during assembly because they can confound De Bruin graph-based assembly of the mt genomic regions in which they are embedded. Recent or well-preserved plastid insertions have a specious connection to the plastid regions they originate from and produce an unresolvable fork in the assembly graph at the junction of a mt-inserted plastid sequence and native mt sequence. Manual assembly, detailed mapping analyses or physical sequencing may be required to confirm their presence.

Plant mt genome dynamics are unique, multifaceted and offer a window into the complex processes of organellar genome evolution, and grasses (Poaceae) are an ideal clade in which to examine mt genome dynamics. The mt genome content of grasses is in flux and numerous reference mt genomes are available in NCBI. Of nine documented transfers to the nucleus from the mt genomes of grasses, *rpl2* (Subramanian and Bonen 2006) and *rpl5* (Sandoval et al. 2004; Wu et al. 2017) are currently undergoing functional transfer, with seven others transferred prior to the origin of the family. Of the seven older transfers, *rpl10* occurred at the base of monocots (Adams et al. 2002; Mower and Bonen 2009; Kubo and Arimura 2010), *sdh3* and *sdh4* occurred subsequent to the origin of the Poales (Adams, Rosenblueth, et al. 2001) and four occurred within the common ancestor of Poaceae, including *rps10* (Adams et al. 2002), *rps11* (Bergthorsson et al. 2004), *rps14* (Sandoval et al. 2004; Ong and Palmer 2006), and *rps19* (Fallahi et al. 2005; Atluri et al. 2015). The progressive loss of a mt sequence may vary dramatically for any given gene; for example, *rps19* was transferred in the common ancestor of *Poaceae*, yet its current distribution and function within grass mitochondria is variable. It is present as a functional copy in *B.inermis*, a pseudogene in *T.aestivum*, and absent altogether from *Hordeum vulgare* (Fallahi et al. 2005; Atluri et al. 2015).

As part of a larger goosegrass [*Eleusine indica* (L.) Gaertn. (Poaceae - Chloridoideae)] genome project (Zhang et al. 2016, 2019), we have undertaken the sequencing of the goosegrass mt genome. Developing mt genomic resources for *E. indica* improves our understanding of the evolution of a seriously invasive and increasingly herbicide resistant grass species (Waldin et al. 1992; Zeng and Baird 1997; Buker et al. 2002) and provides resources for the improvement of an orphan crop plant providing core nutritional support in arid regions of East Africa and West India.

Characterization of complete mt genomes is, presumably, the best way to assess mt gene loss, but in the absence of fully assembled genomes mt gene content is commonly inferred through Southern blot analyses (Palmer et al. 2000; Adams and Palmer 2003). Here, we develop an in silico analog of Southern blotting to investigate gene content in a large sample of Poaceae species for which no mt genome is currently available. Through comparison to our newly sequenced *E. indica* mt genome as well as several other mt genomes available from grasses, we demonstrate that this approach provides results consistent with previous analyses (Palmer et al. 2000; Adams, Ong, et al. 2001; Adams and Palmer 2003; Liu et al. 2009; Atluri et al. 2015; Wu et al. 2017).

## Materials and Methods

### Mitochondrial Genome Assembly

Whole genomic DNA was extracted from fresh leaves of *E.indica* using DNeasey Plant Mini Kit (Qiagen, CA, USA) as part of a genome sequencing project and are available from NCBI. There were two-paired-end libraries SRR7085643 (insert size 200 bp) and SRR7085644 (insert size 400 bp) and one mate-pair library SRR708564 (insert size 7000 bp) (Zhang et al. 2019). We assessed the quality of fastq files with FastQC (Andrews 2010) and used Trim Galore v0.4.0 (Krueger 2015) to remove low quality and adapter sequences; resultant reads were filtered again to remove any reads mapping to plastid or technical sequence (supplementary table 1, Supplementary Material online).

Plant mt genomes are largely composed of highly divergent, intergenic space making impractical most “mapping plus assembly” plastid sequencing pipelines that use a closely related reference genome to create a subset of reads for optimized organellar assembly (Steele et al. 2012; Könyves et al. 2018). However, a similar effect could be achieved in three stages. First we created a large, *Eleusine* specific mt pseudomolecule using low stringency assembly methods [Velvet v1.2.10 (Zerbino and Birney 2008) with VelvetOptimizer v2.2.5 (Gladman and Seemann 2012), Ray v2.0.1 (Boisvert et al. 2010), and AllPaths-LG v3 (Gnerre et al. 2011)], confirming the presence of highly conserved genes within our assembly against *Zea mays* (GenBank accession NC_007982). Second, we assembled a more conservative set of contigs using Ray on the subset of reads derived from the first assembly (focused) as well as the entire set of original reads, in case any mt sequence was dropped during focused mt assembly. Third, we circularized the higher-stringency Ray contigs, preferencing the product of the focused assembly. Our sequence was circularized by generally following methods described by Mower et al. (2012). Contig assemblies were validated by support of mate-pair reads mapping across the length of the entire assembly. Contigs that were misassembled were broken at the junction not crossed by mate-pair and paired end reads. Depth across all mt contigs was calculated, and if a region exhibited approximately twice the depth of an expected mt sequence we inferred that it was duplicated and used the sequence twice for circularization. Given the length of the duplicated regions, we believe that it was reasonable to assume the extra reads were neither plastid nor nuclear in origin because overall mt coverage was well under half that of the plastid genome and the likelihood of having so many (supplementary table 1, Supplementary Material online) insertions so recent or well preserved in the nuclear DNA that they would map across the mt contigs is low. Mitochondrial contigs were then joined if they exhibited overlapping ends, identified with BlastN and concordantly mapping mate pair and paired end reads. Where multiple circular confirmations were possible, the confirmation with the highest number of reads supporting it was used.

To accurately assemble across and validate genuine plastid insertions, a two-step process was employed. First the putative transfer region, from our published *Eleusine* plastid genome (Zhang et al. 2016) was used to scaffold mt contigs based on overlapping ends. Then mated reads, anchored in both the mt sequence and the plastid sequence were used to identify and confirm sequence differences between the plastid inserts in the mt genome and the original plastid genome. To our knowledge, this is first time a focused plastid correction step has been employed to create a pure, mt, version of a plastid insert.

Final assembly was verified by visually confirming a uniform mapping depth of reads mapping across the reference using Bowtie 2 (Langmead and Salzberg 2012). Even with stringent mapping parameters, plastid insertions exhibit slightly elevated coverage where plastid reads are nearly identical to the mt insert reference sequence.

### Annotation, Repetitive Fraction, and RNA Editing

The finalized mt genome sequence was annotated with Mitofy v1.3.1 (Alverson et al. 2010), NCBI BLAST+, and Artemis v16.0.0 (Rutherford et al. 2000). Transfer RNAs were identified using tRNAScan v1.3.1 (Lowe and Eddy 1997) and filtered for length less than 100 and COVE Score >21. RepeatMasker version v3.2.7 (Smit et al. 1996), RepBase version 20090604 (Jurka et al. 2005), and 31 unique sequences with length of >100 were annotated and extracted. These segments were compared with the Reference Mitochondrial Genomes of all angiosperms available on NCBI (accessed October 2016). Tandem repeats and simple sequence repeats (SSRs) were identified using Misa (http://pgrc.ipk-gatersleben.de/misa, accessed December 13, 2016) with a minimum five repetitions for mono and dinucleotide repeats, minimum three repetitions for tri to octa nucleotide repeats, and minimum two repetitions for nona to 31× nucleotide repeats. 

To quantify the extent of mt RNA editing, monoisolate transcriptome reads were downloaded from NCBI SRA (GenBank Accession ERR1590130), checked with FastQC (Andrews 2010), and trimmed for quality with Trimmomatic v.0.3.3 (Bolger and Giorgi 2014). Quality trimming was confirmed with FastQC (Krueger 2015). Two mappings were used to locate potential RNA edits. To search for putative noncoding edits, the finished mt genome and plastid genome (GenBank Accession NC_030486) were concatenated into a single reference. To search exclusively within coding regions, CDS regions were extracted from mt (GenBank Accession MF616338) and plastid (GenBank Accession KU833246) genomes and made into a combined reference. For both noncoding and coding reference files, reads were mapped with Bowtie 2 (Langmead and Salzberg 2012) employing local alignment and match bonus, for genomic mapping and end to end mapping for coding sequences, duplicates were marked with Picard Tools and variants were called with BCF tools v1.5 (https://github.com/samtools/bcftools; last accessed November 20, 2019). C–U edits were counted for coding regions by tallying all C–T variants within mt CDS sequences, and for noncoding regions, C–U edits were tallied C–T or T–C variants on the mt genome after the exclusion of coding region using vcftools v0.1.14-14 (Danecek et al. 2011). Minimum accepted variant depth was set at a frequency of 0.2. Coding sequences for the 33 protein coding genes were extracted from the annotation and 1 copy of each gene was submitted to Prep-Mt with a cutoff of 0.2 (Mower 2005).

### Survey of Poaceae mt Gene Content

Despite the overall profusion of genomic data a concurrent increase in plant mt genome sequences has not materialized. However, mt genome content and evolution is broadly interesting to a range of areas including cytonuclear interactions (Havird et al. 2015), horizontal gene transfer (Bergthorsson et al. 2003), and the evolution of organellar genomes (Palmer et al. 2000). Here we develop an approach to leverage whole genomic SRA data to address basic questions in plant mt genomics. This method is used to examine conserved mt genes across the breadth of Poaceae, establishing gene presence in the mt genome and producing a quality assembly of the gene sequences for downstream analyses (supplementary fig.1Supplementary Material online).

To visualize the relative abundance and copy number of mt genes a heat map and a coverage plotting approach were deployed in tandem. Heat map color was assigned based on read coverage relative to the average mapping depth (calculated in SAMtools v1.2, Li et al. 2009) of three large and consistently mt-located control genes. Scoring ranges from absent (black) to ultrahigh-coverage (light yellow or white), with loci lacking 50% coverage >5 automatically scored as absent. Coverage plots for a subset of genes was constructed with the *n*-fold + 1e−5 mt depth transformed with the natural log and plotted across the length of the gene. One control gene, *matR*, was plotted as a visual reference and variable regions *rpl2*, *rpl5*, *rpl10ψ*, *rps11*, *rps14*, and *rps19* were plotted to determine the pattern of coverage with Python 3.4.3 with Matplotlib v1.3.1 (Hunter 2007) and Pandas v0.21.0 (McKinney 2010) libraries. Coverage plotting was also applied to a subset of taxa for the genomic region around *rpl5* and *rps14* genes to determine if they were undergoing tandem loss. Mt gene complement was determined for *Alloteropsis cimicina* (GenBank accession SRR2163548), *Aristida congesta* (GenBank accession SRR2163568), *Aristida purpurea* (GenBank accession SRR2163569), *Danthoniopsis dinteri* (GenBank accession SRR2163566), *Echinochloa frumentacea* (GenBank accession SRR2162759), *E.indica*, *Eragrostis tef* (GenBank accession SRR1463402), *Leersia perrieri* (GenBank accession SRR1528439), *Oropetium thomaeum* (GenBank accession SRR2083764), *Oryza longistaminata* (GenBank accession SRR1264538), *Oryza punctata* (GenBank accession SRR1264539), *Sporobolus michauxianus* (GenBank accession SRR486071), *Triticum monococcum* (GenBank accession SRR445609), *Triticum turgidum* (GenBank accession ERR463920), *Triticum uratu* (GenBank accession ERR424867), and a 10,044 bp region of *Oryza sativa* (GenBank accession NC_007886.1: 340483–350527) containing the 3′ end of cox1, intergenic spacers, *rps14*, and *rpl5*, using the *Z.may*s *matR*, *nad7*, and *nad4* sequences as controls for depth normalization.

We employed a consensus method to extract sequences of each mt gene for phylogenetic analysis. This allowed us to include genes with sparse coverage that would not have assembled using a short read assembler. Fastq files were mapped to plastid and mt reference sequences using Bowtie 2 (Langmead and Salzberg 2012) local alignment with match bonus, qc-filter and up to 900 M reads). BAM files were filtered to exclude reads that mapped to plastid references, converted to fastq files and processed with Trim Galore (Krueger 2015) using the “Illumina” flag to identify known adapter sequences. Filtered reads were mapped back to the mt reference using the original parameters, resulting maps were filtered with cigar_filter.py with minimum match length of 25 (https://github.com/NDHall/pysam_tools/tree/master/cigar_filter; last accessed November 20, 2019), and passed through the best practice Picard and GATK pipeline to the realignment step (Van der Auwera et al. 2013) using GATK v3.6 (McKenna et al. 2010). Realigned BAM files were used to produce reference-free consensus sequences using pysam_consensus.py and a minimum average depth of 6 (https://github.com/NDHall/pysam_tools/tree/master/consensus_caller; last accessed November 20, 2019). A modified version of fasta-stats.py (https://techoverflow.net/2013/10/24/a-simple-tool-for-fasta-statistics/, accessed July 20, 2017) was used to produce input for fasta_stats_parser.py

(https://github.com/NDHall/pysam_tools/tree/master/fasta_stats; last accessed November 20, 2019) with default settings which limited sequences to high-quality, high-coverage consensus sequences for broader Poaceae to produce a list of acceptable assemblies which were extracted with select_contigs.pl (https://github.com/chrishah/phylog/blob/master/scripts-external/select_contigs.pl; last accessed November 20, 2019) and aligned in Mafft v7.123 (Katoh and Standley 2013). Alignments were visually inspected and set in-frame using Seaview v4.0 (Gouy et al. 2010). Individual taxa were retained for downstream analyses if they had data for 10,000 nucleotide positions (of 27,848 possible) or more, and each alignment was ordered and expanded to include missing taxa using fasta_ghost.py (https://github.com/NDHall/pysam_tools/tree/master/fasta_ghost; last accessed November 20, 2019) and concatenated using FASconCAT v1.02 (Kück and Meusemann 2010). Final sequences were spot checked against known reference genomes using the BLAST search utility on NCBI.

### Phylogenetic Analysis and Rates Analyses

We wanted to explore the utility of assembled sequences for informing a reference phylogeny and examining mt genome rate variation within the chloroidoid clade that contains *Eleusine*. To construct the backbone phylogeny, a full codon by gene partition scheme was examined with Partition-Finder v2.0.0 (Lanfear et al. 2012), model selection limited to GTR-GAMMA and GTR-GAMMA+I with greedy search algorithm, and the best scheme subsequently used for phylogenetic analysis. Trees were created using RAxML-MPI-AVX v8.2.6 (Stamatakis 2014) with 100 rapid bootstraps, GTRGAMMA model and the best partition scheme returned by Partition-Finder. Trees were visualized with FigTree v1.4.1 (Rambaut 2009). To explore individual rates of gene evolution for chloroids, *E.indica* (GenBank accession SRR7085643), *S.michauxianus* (GenBank accession SRR556090), and *O.thomaeum* (GenBank accession SRR2083764) were included in estimates of nucleotide substitutions made by Mega-cc v7.0.2 (Kumar et al. 2012) and PAML v4 (Yang 2007). Nucleotide alignments were created with Clustal v1.2.1 (Thompson et al. 1994). All sites containing gaps were excluded from the analysis and rates of substitution were compared among subunits. Codeml from PAML 4.9b (Yang 2007) was used to determine d*N* and d*S* values for subunits with high substitution rates (i.e., *atp1*, *atp4*, *atp6, atp8*, *atp9*) for chloroids with *Ananas comosus* (GenBank accession DRR022930) as the outgroup. Sequences were taken from consensus sequences prepared as before and gene alignments were created with codon aware Mega-cc (Kumar et al. 2012), visualized and curated with Seaview (Gouy et al. 2010). Codeml was run for chloroid sets with free rates, ambiguous sites removed, a starting κ of 2.0 and a starting ω of 0.4 (supplementary material 1, Supplementary Material online).

## Results

### Mitochondrial Genome Assembly and Annotation

**Fig. 1.**
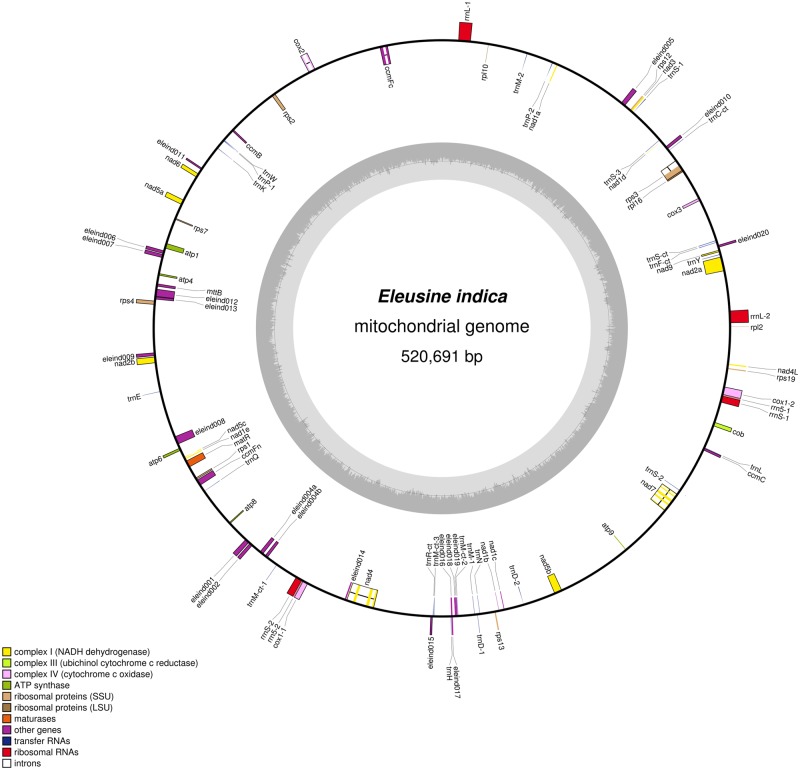
—Map of circularized *Eleusine indica* mitochondrial genome. Genes marked on the outside of the circle run counterclockwise, genes marked on the inside run clockwise. Color is assigned based on function and hypothetical proteins are marked “eleind.” The inner circle shows GC (dark gray) and AT (light gray) content.

**Fig. 2.**
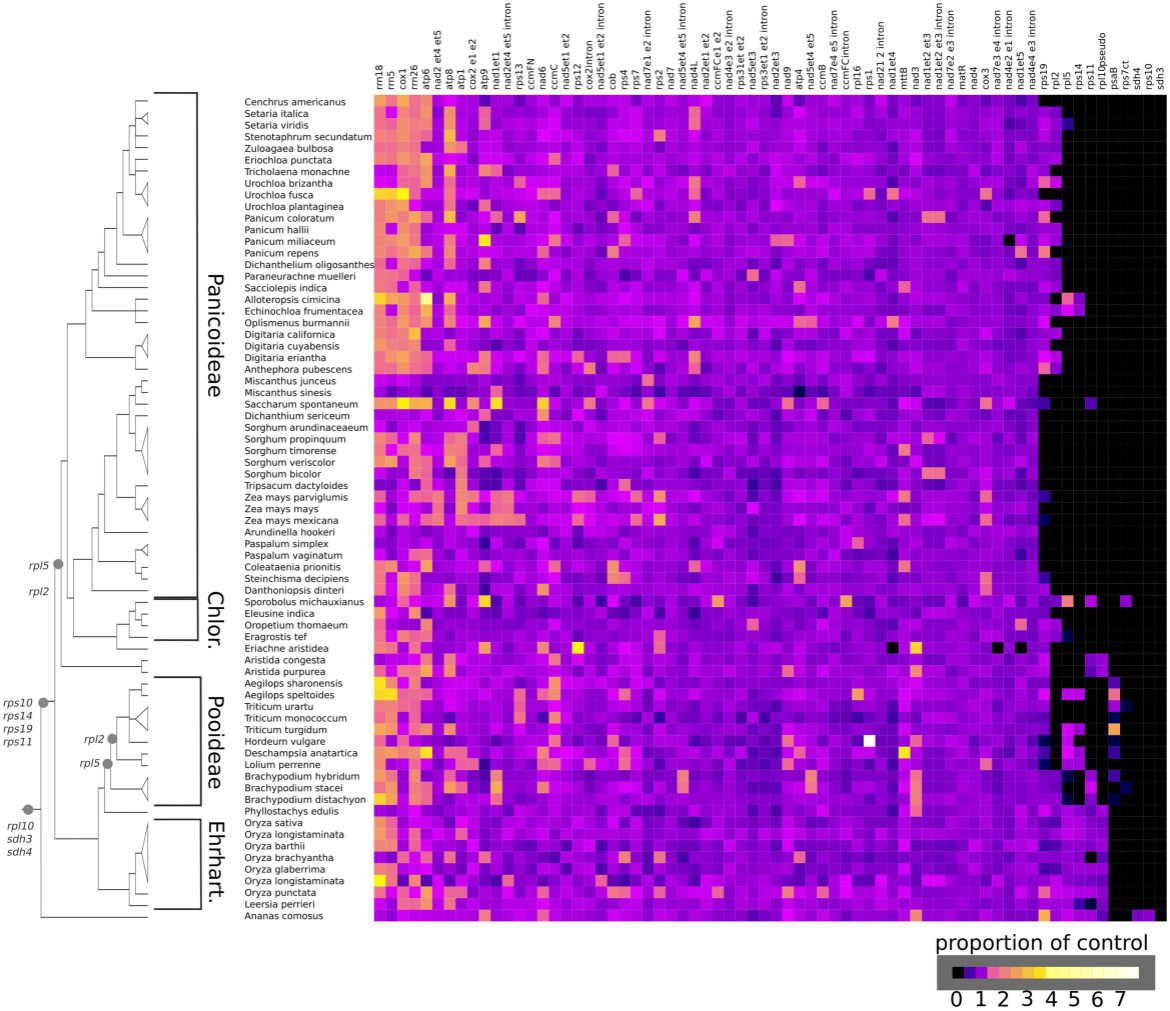
—Read depth-based heat map showing the variable intensity or lack of mt gene signature in Poaceae and *A. comosus* SRA readsets. Phylogenetic organization is taken from GPWG (2011). Functional transfer of genes to the nucleus indicated with gray dots: s*dh3, sdh4* (Adams, Rosenblueth, et al. 2001), *rpl10* (Mower and Bonen 2009; Adams et al. 2002; Kubo and Arimura 2010), *rps10* (Adams et al. 2002)*, rps11* (Bergthorsson et al. 2004), *rps14* (Sandoval et al. 2004; Ong and Palmer 2006), *rps19* (Fallahi et al. 2005; Atluri et al. 2015), *rpl2* (Subramanian and Bonen 2006), and *rpl5* (Sandoval et al. 2004; Wu et al. 2017). The *rpl2* transfer is placed in a deeper position in PACMAD clade than previously reported (Subramanian and Bonen 2006) based on the uniform absence of an intact orf in descending lineages (supplementary fig. 5, Supplementary Material online).

Sequencing of the three DNA libraries, SRR7085643, SRR7085644, and SRR7085644, yielded 202 million reads, reduced to 178 million after filtering and quality control (supplementary table 1, Supplementary Material online). The final, circularized *E.indica* mt genome was 520,691 bp (GenBank accession MF616338) representing 3% of the 178 million filtered reads. Uniform and complete coverage of the master circle was observed by concordant mapping of paired end and mate pair reads (supplementary fig. 2*a*, Supplementary Material online). Paired end read coverage was somewhat more variable but exhibited a depth of ∼400 reads per nucleotide, per read set across portions of the genome unique to the mitochondria (supplementary table 1 and fig. 1*b*, Supplementary Material online) and >8,000 reads/nt for portions of the mt genome containing plastid transfer sequence, even with our mt correction technique (supplementary fig. 2*b*, Supplementary Material online). Mate-pair read coverage was less variable but exhibited the same pattern with unique mt sequence having a mean coverage of 40 reads per nucleotide (supplementary table 1 and fig. 2*b*, Supplementary Material online).

The mt genome of *E.indica* contained a total of 33 mt protein coding genes, 2 pseudogenes (*rpl2*, *rpl10*), 6 rRNA (2 copies each of 3 subunits), and 24 total tRNA with 15 unique tRNAs (fig. 1) were annotated. Twelve separate plastid transfers >400 bp were detected, representing multiple ORFs (*psaA*, *psaB*, *rpl2-ct*, *Ycf2*, *atpA*, *ndhJ*) and 9 tRNA of apparent plastid origin. Eight large repetitive regions >400 bp were identified with BlastN and designated A–H. Two of the repeat regions contained genes (A: *rrnL*, B: *rpl2ψ*): and two contained retroelements (B, C). Repeatmasker identified several transposable elements within the mt genome accounting for more than 15 kb of sequence, or 2.9% of the total mt genome. Misa identified a total of 3,209 SSRs, a majority of which were mononucleotide repeats (2,820) and nearly one-third of the repeats were in compound formation (1,630) (supplementary table 2, Supplementary Material online). A total of 620 RNA edit sites were predicted, 530 of these in coding regions, 90 in noncoding regions (supplementary table 3, Supplementary Material online), with a majority of edits occurring in first and second codon positions (supplementary table 4, Supplementary Material online).

### Phylogenetic Analysis and Rates Analyses

A total of 114 SRA data sets were filtered and mapped to mt reference exons resulting in 3.3 M reads input to the consensus-calling pipeline. Consensus calling, alignment and filtering produced 71 data sets that were combined in a supermatrix that was 74.08% complete with 3,138 distinct alignment patterns. RAxML (Stamatakis 2014) analyses consistently recovered a Chloroid clade, correctly placing *Eleusine* sister to *Oropetium* and *Sporobolus* sister to *Eleusine* and *Oropetium*. Internal nodes of the overall Poaceae phylogeny were weakly supported and incongruent with accepted relationships, for example, the *Oryza/Leersia* clade was placed incorrectly and there was poor resolution for undersampled portions of the PACMAD clade (supplementary fig. 3, Supplementary Material online). Rate analysis with Mega revealed relatively low rates substitution ranging from 1.53× 10^−4^ to 3.54×10^−3^ for all mt-encoded gene subunits (*nad*, *mtt*, *rpl*, *cob*, *cox*, *mat*, *ccm*, *rps*, and *atp*) (supplementary table 5, Supplementary Material online). Follow up with codeml in PAML showed that the fastest category of genes, those composing the ATPase subunit were driven by synonymous substitutions [d*S*: mean = 0.138, standard deviation (SD) = 0.0976, max = 0.4316, min = 0.056; d*N*: mean = 0.0404, SD = 0.0327, max = 0.109, min = 0.0131] (supplementary material 2 and fig. 4, Supplementary Material online). d*S* showed elevated rates of substitution in *atp8* [*S.michauxianus* (GenBank accession SRR556090) = 0.1536, *O.thomaeum* (GenBank accession SRR2083764)=0.1716, *E.indica*=0.1717] and *atp9* [*S. michauxianus* (GenBank accession SRR556090)=0.4316, *O. thomaeum* (GenBank accession SRR2083764)=0.1718, *E.indica *=* *0.2394], and d*N*/d*S* ratios as a function of subunit showed elevated ratios in *atp4* [*S. michauxianus* (GenBank accession SRR556090)=0.744, *O. thomaeum* (GenBank accession SRR2083764)=0.6982, *E. indica *=* *0.638], and *atp8* [GenBank accession *S. michauxianus* (SRR556090)=0.637, *O. thomaeum* (GenBank accession SRR2083764)=0.636, *E. indica = *0.542].

### Survey of Poaceae mt Gene Content

**Fig. 3.**
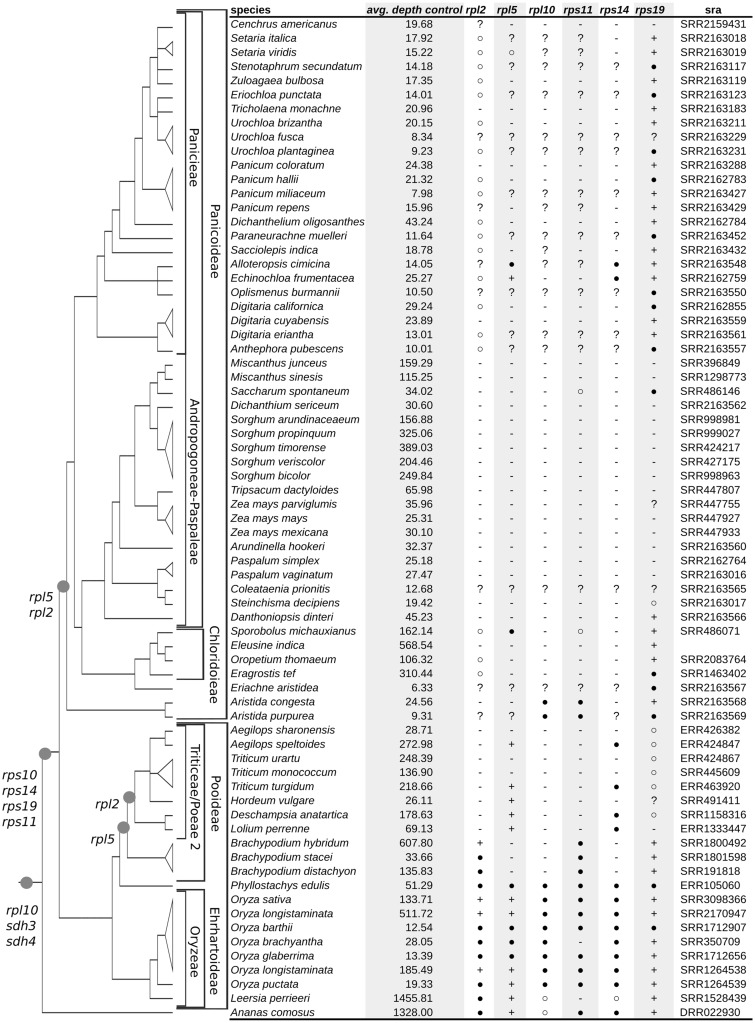
—Summary of high confidence mt gene presence/absence calls. Presence calls grouped into three tiers based on overall read support for each sequence. Third tier (○) genes have an average heat map value above 0.25. Second tier (●) genes have continuous coverage across the full length of the gene with logarithmic score greater than ca. −2.0, that is, *n*-fold 0.135 coverage (supplementary fig. 5, Supplementary Material online). First tier (+) genes possess sufficient depth of coverage to assemble an intact reading frame. Absence (−) called for low to no coverage of mt sequence where average control is otherwise >15. Ambiguity (?) related to low mitochondrial sequencing depth overall in readset. Grass phylogeny and functional transfers follow figure 2.

**Fig. 4.**
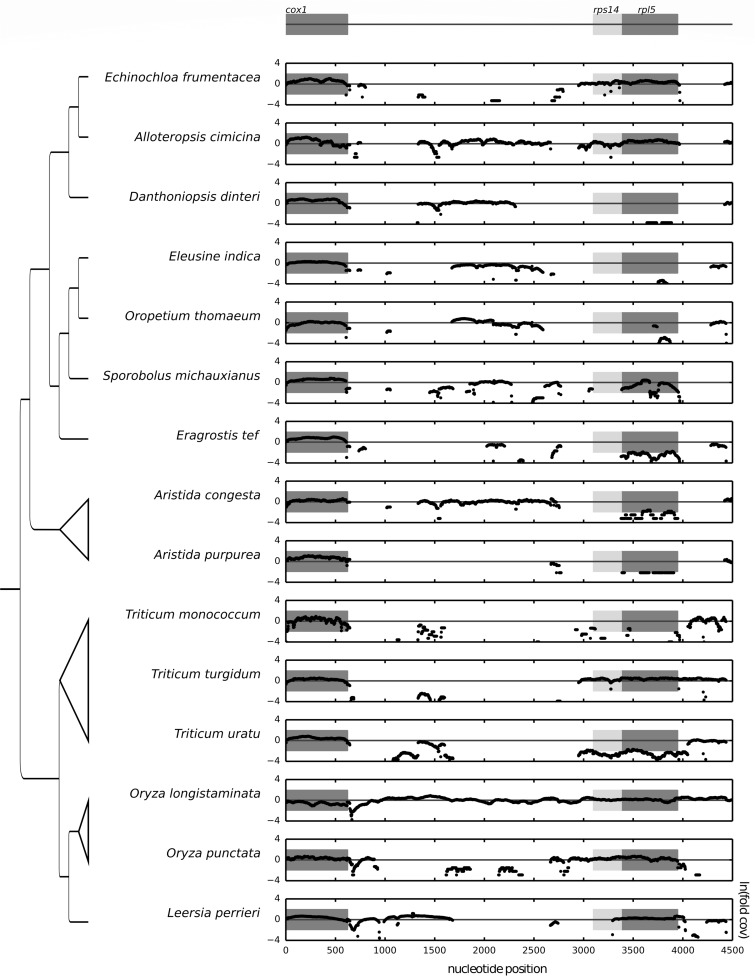
—Natural log of mt normalized read depth plus 1e−10. The universal mt gene *cox1* serves as the positive control and *rps14* and *rpl5* are variable. Gray boxes mark the borders of the gene on the *x* axis for the reference sequence (*Oryza sativa, NC_007886.1: 340483–350527*); the *y* axis boundaries represent the natural log of a 6-fold deviation from the expected mt coverage, distinguishing mt and nuclear sequences. For example, *Triticum uratu* is deeply sequenced and nuclear-encoded genes are visible and must be distinguished from the mt sequence.

There are several functional mt gene transfers placed prior to the origin of the Poaceae (*rpl10*, *rps10*, *rps11*, *rps14*, *rps19, sdh3*, and *sdh4*) and two apparently ongoing transfers within the family (*rpl2* and *rpl5*). Broadly scoring the presence/absence of these variable genes reveals an infrafamilial mosaic of mt gene transfer and loss because even a functional transfer to the nucleus is not immediately concurrent with loss of mt sequence. Furthermore, it has the potential to highlight rare cases of gene regain (Brennicke et al. 1993; Adams and Palmer 2003; Bonen 2006). Using available SRA data, we examined the presence/absence of mt genes in 70 species of Poaceae and 1 Bromeliad through analysis of read depth (figs. 2 and 3; supplementary fig. 5, Supplementary Material online). The resulting heat map shows diverse patterns of lineage-specific loss over time in grasses. Most genes, particularly those involved in oxidative phosphorylation, have been retained in all lineages, consistent with previous studies (Adams et al. 2002; Adams and Palmer 2003). In contrast, several genes (*rps10*, *sdh3*, and *sdh4*) are absent from all Poaceae species, indicating that they were lost prior to the origin of grasses. Six ribosomal protein genes (*rpl2*, *rpl5*, *rpl10*, *rps11*, *rps14*, and *rps19*) exhibit a varied pattern of differential retention and loss across Poaceae phylogeny (figs. 3 and 4).

To verify the accuracy of the heat map analysis in determining the presence or the absence of genes in the mt genome, we compared the results of that pipeline with our sequenced *E.indica* mt genome, and to eight other Poaceae mt genomes available in GenBank (*Aegilops speltoides*, *H.vulgare*, *Lolium perenne*, *O.sativa*, *Sorghum bicolor*, *Tripsacum datcyloides*, *Z.mays* subsp. *mays*, and *Z.mays* subsp. *parviglumis*). The heat map confirms that all nine species lack the *rps10*, *sdh3*, and *sdh4* genes, as expected. Among the six variably present ribosomal protein genes the heat map approach agrees with previously reported results. The heat map suggests that *Sorghum*, *Tripsacum*, and *Zea* have lost all six, *E.indica* has retained *rps19*, *Hordeum* has retained *rpl5*, *Lolium* has retained *rpl5* and a large *rps14* pseudogene, *Aegilops* has retained *rpl5* and two large pseudogenes (*rps14* and *rps19*), and *Oryza* has retained *rpl2*, *rpl5*, *rps19*, and three large pseudogenes (*rpl10*, *rps11*, and *rps14*). The heat map analysis failed to detect several smaller (<100 bp) pseudogenes of *rpl10* and *rps19* in a few species, suggesting an approximate lower limit of detection for this approach.

More broadly, the heat map analysis, coupled with normalized read depth visualization and inspection of assembled gene sequences, suggests frequent loss of coding regions, particularly associated with *rps19, rpl2*, and *rpl5*, across Poaceae (fig. 3). The results of coverage plotting, by gene, heat map, and sequence assembly show broad patterns of loss over time. The depth plots show the successive loss of r*pl2* sequence within PACMAD clade and Pooideae in the BEP clade, as different portions of the pseudogenized sequence have been lost over time (supplementary fig. 5, Supplementary Material online). The occurrence of *rpl5* has been well characterized in the both PACMAD and BEP clades of Poaceae and additional losses in *Setaria*, *Cenchrus*, *Eleusine*, and *Panicum* are evident here. The assembled consensus sequences for *rpl5* strongly suggest pseudogenization in *Sporobolus* and *Echinochloa*, and the likely retention of a functional copy within *Alloteropsis*. The variable loss of *rpl5* from the mitochondria within Paniceae is striking, because it appears that both *Alloteropsis* and *Echinochloa* have retained mt copies of the gene, whereas *Oplismenus* has lost its copy. The pseudogene *rps14* seems to show a similar pattern as *rpl5* within *Alloteropsis* and *Echinochloa*, and it is likely that the fates of the two physically linked genes are related.

The loss of *rps19* from within the BEP portion of Poaceae has been studied extensively (Atluri et al. 2015). Our results concur with those previously reported and broaden the perspective of *rps19* loss and retention within PACMAD clade. Coverage depth plots of *rps19* across the PACMAD clade suggest only Andropogoneae, Arundinelleae, and Paspaleae have experienced widespread loss of this gene. Consensus sequence of *rps19* strongly suggests it persists as a mt functional copy within Aristidoideae, Chloridoideae, and Paniceae. Within PACMAD clades possessing *rps19*, multiple, independent losses suggest its eventual fate. For example, within Paniceae, *Cenchrus* shows evidence of having completely lost *rps19* from its mt genome, the first reported loss for *rps19* within Paniceae. The *rps11* pseudogene has been lost across PACMAD clade, and within Triticeae and Poeae 2 clades of BEP. Furthermore, there appears to be a single, recent, novel loss of *rps11* sequence within *Oryza brachyantha*, uniquely significant given that economically important genus.

Our analysis also points to an older age for the *rpl2* transfer associated with the PACMAD clade. Examination of read depths across *rpl2* containing PACMAD taxa shows a range of patterns from the most intact loci with minor deletions in exon 1 or the group II intron (Subramanian and Bonen 2006) to the complete absence of any recognizable *rpl2* sequence (supplementary fig. 5, Supplementary Material online). Assembly of a representative deeply sequenced sample, *O.thomaeum* (GenBank accession SRR2083764), produces a single contig with structural similarity to the *rpl2* pseudogene from *B.inermis* (GenBank accession KT022083.1). The apparent pseudogenization of *rpl2* in all surveyed PACMAD sequences suggests a common transfer of *rpl2* at the base of PACMAD clade. Similar patterns of loss were evident for Poaeae 2 and Triticeae consistent with previous reports (Subramanian and Bonen 2006).

## Discussion

The *Eleusine* mt genome exhibits a Poaceae-typical set of mt genes, with foreign DNA from the plastid and nucleus and several recombinogenic repeats that promote a multipartite structure. Mt genes performed weakly in phylogenetic analyses, not surprising given the long-known degree of sequence conservation (Palmer and Herbon 1988). Investigation of the rates of individual gene complexes suggest a pattern of low substitution rates for *nad* genes and accelerated rates for *atp* and *cox* genes following the broad pattern of sequence evolution described by Cui et al. (2009). In the case of the chloroidoid grasses examined here, increased rates involve increased neutral substitutions as described and elsewhere and no species exhibited particularly high rates of substitution (Cui et al. 2009). In summary, our results suggest that *E.indica* has an average mt genome for the Poaceae.

### Survey of Poaceae mt Gene Content

Visualizing copy number of putative mt sequences in silico is a potentially broadly useful tool for plant mt genome analysis. Our results for gene content closely match those reported within the literature, with a few discrepancies that in fact provide further insight into the gene content and evolutionary dynamics within mt genomes of Poaceae. There are apparent cases of ongoing losses within species such as *D.dinteri*, in which a pseudogene for *rpl5* was reported (Wu et al. 2017, supplementary material online) and a pseudogene was reported for *rps14* (Ong and Palmer 2006), whereas the sampling of SRA data (GenBank accession SRR2163566) suggests that both *rpl5* and *rps14* are missing entirely from the individual sampled (figs. 3 and 4; supplementary fig. 5, Supplementary Material online). Wu et al. (2017) report that *rpl5* is likely missing from *Triticum urartu*; our results concur and have the added benefit of showing that it is indeed absent from the mt genome by virtue of its low depth of coverage (GenBank accession ERR424867). Additionally within *Triticum*, *T. turgidum* (GenBank accession ERR463920) is an AABB tetraploid. The AA genome donor was most likely *T.uratu* (Petersen et al. 2006) and the putative BB genome donor was *A.speltoides* (GenBank accession ERR424847; Miki et al. 2019). It appears that the *rps14* and *rpl5* were lost from *T. uratu* prior to the hybrid formation of *T. turgidum* because these genes are also missing from *T. monococcum* (GenBank accession SRR445609), sister to *T. uratu* (Michikawa et al. 2019), in contrast to their confirmed presence in *A. speltoides* (figs. 4). Taken together these observations suggest that *rps14* and *rpl5* were lost in the common ancestor of *T. uratu* and *T. monococcum* prior to the formation of *T. turgidum* and that the extant copies of *rps14* and *rpl5* within *T. turgidum* are from the polyploidization event with *A. speltoides*. Additional study is warranted in all of the above systems to explore these novel hypotheses of mt gene content evolution.

The in silico analyses reveal other patterns of mt gene loss infrequently examined at this scale within Poaceae. The genes *rpl5* and *rps14* are tightly linked within virtually all angiosperms (Ong and Palmer 2006) and as a consequence their patterns of loss are frequently linked as well (figs. 4). Notable exceptions are *S.michauxianus* (Genbank accession SRR486071) and *L.perrieri* (Genbank accession SRR1528439) in which *rps14* has been lost independently of *rpl5.* The loss of *rps14* in the context of its ancestral spatial relationship relative to *rpl5* provides further insight into the rate and scale of sequence loss within plant mt genomes. Two other pseudogenized sequences, *rps11* and *rpl10*, are sparsely reported on within Poaceae but also provide insight into the heterogeneous loss of mt sequence. The *rpl10* and *rps11* pseudogenes are generally present within BEP clade absent from the PACMAD clade, but there are a few exceptions to this pattern that can be found within our sampling. The loss of *rps11* in *O.brachyantha* (Genbank accession SRR350709) appears to have occurred after the divergence of the FF *Oryza* genome from the rest of *Oryza* (Nishikawa 2005). The *rpl10* pseudogene is present in *Aristida* yet absent throughout the rest of the PACMAD clade samples, suggesting that it was lost subsequent to the divergence of *Aristida* from all other PACMAD taxa. Finally, these data provide deeper insight into the functional transfers of *rpl2* within the PACMAD clade. It is intriguing that the *rpl2* nuclear transfers are from different events, yet the pseudogene sequences share strong similarity to the *B.inermis* pseudogene that has specific deletions in exon 1 and the group II intron (Subramanian and Bonen 2006).

### Potential Limits of the In Silico Approach

Our method has allowed for a previously unseen, large scale visualization of mt gene loss in line with previous findings, but there are natural limits imposed by the read mapping approach and basic assumptions about the accuracy of the reference sequences used. Read mapping allows the highly sensitive detection of gene presence but is an unreliable indicator of gene absence in organisms with low mt genomic coverage—often key species most needed to fill in gaps in our understanding. In a similar vein, there is a lower limit to mapping resolution imposed by length of the reference sequence. An extremely short reference sequence (e.g., <20 bp) will rarely if ever produce a high enough mapping score to pass QC thresholds and thus must be excluded from consideration for this method. A potential solution for closely related groups of species would be to add flanking sequence to each short target exon, with the obvious limit imposed by phylogenetic distance between reference and target read set.

Comparisons to a predefined lineage assume that the underlying reference genome is an accurate representative sequence. This is not always the case (Sloan et al. 2018) and for many taxa such as *E.indica* here, reference genomes are derived from a single line or plant that is assumed to represent an entire species, but may in fact be abnormal. This is a real concern for species such as *D.dinteri* (Wu et al. 2017, fig. 3, supplementary fig. 5, Supplementary Material online) which seems to harbor multiple mitotypes. The only remedy for this problem is to expand sampling of a target clade or taxa. A related concern arises in identifying missing genes, where it is necessary to use a reference from a different and possibly distantly related species that possess the gene. Given the low rate of mt evolution and the generally permissive mapping parameters employed, we had no difficulty using *Liriodendron tulipifera* as a reference for the Poaceae genes *sdh3* and *sdh4* but there could be cause for concern when considering mt genomes with high rates of substitution such as those encountered in *Viscum* (Zervas et al. 2019). Finally, the reliance of this method on control genes assumes they are single copy within the mt genome across all mt sampled. Although this seems to generally be the case, there are instances such as in cytoplasmic male sterile lines where mt subgenomic stoichiometry is not constant, and could conceivably change the mt normalization value (Janska et al. 1998). We believe that our method is robust to minor shifts in mt gene stoichiometry because it spans three loci and because mt genomic sequence is generally present at a high enough rate to successfully differentiate mt sequence from nuclear sequence. Work by Straub et al. (2012) showed that mt sequence was present at an average of 94.3 ×*N* = 14 (minimum 33.3, maximum 185.7) greater than the nuclear genome and 11.6× (minimum 5.08, maximum 25.4) less than plastid genome. Given these numbers, the stoichiometric shifts in mt genome would have to be extreme to confound the ability of mt normalization factor to differentiate between mt and nuclear sequence. The real strength of this method is that it can be employed rapidly, on increasing and freely abundant data, highlighting interesting cases and generating hypotheses to be tested with further work.

## Conclusion

We presented a broad overview of mt genome gene loss across the Poaceae, gleaning information from SRA data sets for grasses with no published mt genomic sequence. This approach allows us to determine patterns of gene loss without full mt genome assembly or tedious laboratory work and includes loci that are rarely studied such as *rpl10* and *rps11* with little extra effort compared with traditional methods. The *E.indica* mt genome and the broader context of gene loss in Poaceae represent a significant step toward a fully genomic understanding of a prolific herbicide resistant weed and genome donor of an important human food source. The analytical approach and tools described here will have wide applicability to similar questions of mt genome evolution throughout plants.

## Data Availability

Alignments are available from https://github.com/NDHall/EleusineMitochondria.

## Supplementary Material

Supplementary data are available at *Genome Biology and Evolution* online.

## Supplementary Material

evz229_Supplementary_Data

## Notes

**Data deposition**: mt sequence is available from NCBI accession MF616338.
