# Supplementary material for: The Mitochondrial Genome of Eleusine indica and Characterization of Gene Content within Poaceae
Source: Genome Biol Evol. 2019 Oct 23;12(1):3684–97. doi: 10.1093/gbe/evz229 (PMC7145533; doi:10.1093/gbe/evz229)
Supplement: evz229_Supplementary_Data [file evz229_supplementary_data.zip › SupMat1-2.pdf]

Supplemental Material 1: PAML flags used to calculate dN and dS values.

var	value	
aaDist	0	
alpha	0	
cleandata	1	
clock	0	
CodonFreq	2	
fix_alpha	1	
fix_blength	0	
fix_kappa	0	
fix_omega	0	
fix_rho	1	
getSE	1	
icode	0	
kappa	2	
Malpha	0	
method	0	
Mgene	1	
model	1	ω (Goldman and Yang 1994; Yang and Nielsen 1998)
ncatG	3	
noisy	9	
NSsites	0	
omega	0.4	
RateAncestor	0	
rho	0	
runmode	0	
seqtype	1	
Small_Diff	5.00E-07	
verbose	1	

Supplemental Material 2: dN and dS values calculated with PAML.

Species	gene	dN	dS	dt	dN/dS
Eleusine indica		0.0179	0.1123	0.1240	0.1594
Oropetium thomaeum (SRR2083764)	atp1	0.0161	0.1031	0.1130	0.1562
Sporobolus michauxianu s (SRR556090)		0.0161	0.1000	0.1107	0.1610
Eleusine indica		0.0474	0.0743	0.1616	0.6380
Oropetium thomaeum (SRR2083764)	atp4	0.0391	0.0560	0.1295	0.6982
Sporobolus michauxianu s (SRR556090)		0.0418	0.0562	0.1358	0.7438
Eleusine indica		0.0255	0.0892	0.1232	0.2859
Oropetium thomaeum (SRR2083764)	atp6	0.0233	0.0607	0.0974	0.3839
Sporobolus michauxianu s (SRR556090)		0.0255	0.0751	0.1126	0.3395
Eleusine indica		0.0931	0.1717	0.3324	0.5422
Oropetium thomaeum (SRR2083764)	atp8	0.1092	0.1716	0.3698	0.6364
Sporobolus michauxianu s (SRR556090)		0.0979	0.1536	0.3314	0.6374
Eleusine indica		0.0131	0.2394	0.2258	0.0547
Oropetium thomaeum (SRR2083764)	atp9	0.0131	0.1718	0.1702	0.0763
Sporobolus michauxianu s (SRR556090)		0.0262	0.4316	0.4093	0.0607
mean		0.0404	0.1378	0.1964	0.3716
std		0.0327	0.0976	0.1085	0.2556
max		0.1092	0.4316	0.4093	0.7438
min		0.0131	0.0560	0.0974	0.0547
