# Supplementary material for: The Mitochondrial Genome of Eleusine indica and Characterization of Gene Content within Poaceae
Source: Genome Biol Evol. 2019 Oct 23;12(1):3684–97. doi: 10.1093/gbe/evz229 (PMC7145533; doi:10.1093/gbe/evz229)
Supplement: evz229_Supplementary_Data [file evz229_supplementary_data.zip › Supplementary_Tables.pdf]

Supplementary Table 1. Read set summary statistics.

**Summary**	**Facility**	**Insert size (bp)**	**Fastq Stats**			**Trim_galore flags**	**Filter Sequences**	**Mean Quality Scores**	**Mapped Reads**		**Mapping Depth**		
			Raw Count	*Filtered Count*	*Read Length After QC (bp)*				*Total*	*Edit distance of 0*	*mean*	*median*	*std*
	SRR7085643	200	91,460,774	75,619,130	75-100	--paired --adapter2 TTCTTTCCCCCACCCTTTCC --length 75	KU666001.1	36.44	3,317,708	2,870,544	5571	440.0	440.0
	SRR7085644	400	100,277,890	82,788,746	90-101	--paired --illumina --length 90	NC_030486	36.92	2,139,526	2,031,907	416	416.0	118.8832
	SRR7085644	7.0e4	110,263,211	21,181,365	90-101	--paired --illumina --nextera	NC_030486	37.09	218,616	20,1672	40	41.2	14.38 67.1
**Total Paired End**			191,738,664	158,407,876									
**Total combined**			202,001,875	179,589,241									

* Longer version (MTV7) of sequence was used to include more reads, due the circular nature of the sequence this allows reads to mapped that would otherwise be cut off.

Supplementary Table 2. Summary of Misa results

Unit Size	No. SSRs
1	2820
2	21
3	270
4	53
5	16
6	3
9	9
10	3
11	1
12	3
14	1
15	2
17	1
20	1
22	1
24	1
26	1
27	1
30	1

Supplementary Table 3. Total unique calls is a list of non-overlapping C to T calls made with alignment of extracted coding sequences mt-prep and bcf calls, allowing for a minimum depth of 1 and a minimum alternate allele frequency of 0.20. Well supported alleles include, all predictions shared between mt-prep and bcf calls, all bcf calls with a depth greater than 10 and a minimum alternate allele frequency of 0.20 and all unique mt-prep calls unsupported by mapping if mapping depth was less than or equal to 10. *The gene eleind009 was not submitted to mt-prep for analysis.

sites	coverage categories	total count	counts
all unique calls		530	
	Depth of site coverage > 10		87
	0 < Depth of site coverage <= 10		373
	Depth of site coverage = 0		70
all mt-prep		426	
	Depth of site coverage > 10		70
	0 < Depth of site coverage <= 10		286
	Depth of site coverage = 0		70
all bcf calls		380	
	Depth of site coverage > 10		75
	0 < Depth of site coverage <= 10		305
	Depth of site coverage = 0		0
all shared		276	
	Depth of site coverage > 10		58
	0 < Depth of site coverage <= 10		218
	Depth of site coverage = 0		0
unique bcf		104	
	Depth of site coverage > 10		17

	0 < Depth of site coverage <= 10		87
	Depth of site coverage = 0		0
unique mt prep		150	
	Depth of site coverage > 10		12
	Depth of site coverage < d <= 10		68
	Depth of site coverage = 0		70
**total well supported**		**431**	

Supplementary Table 4. Well supported RNA editing calls(as defined in Supplementary Table 3) compared with all calls, the total set of unique calls. Calls are compared by gene and codon position. Average depth calculated by dividing sum of depth for all mapped positions by length of the gene. *Not submitted to mt-prep for analysis.

**category**	**avg. depth**	**counts per gene**		**codon**	**counts per codon**	
		**well supp.**	**all**		**well supp.**	**all**
atp1	26.78	6	7			
				**2**	4	5
				**3**	2	2
atp4	4.59	9	10			
				**1**	2	2
				**2**	7	8
atp6	4.47	14	17			
				**1**	4	4
				**2**	10	10
				**3**		3

atp8	14.32	6	6			
				**1**	3	3
				**2**	2	5
				**3**	1	2
atp9	3117.54	7	7			
				**1**	1	1
				**2**	6	6
ccmB	0.24	31	31			
				**1**	12	12
				**2**	19	19
ccmC	3.10	36	40			
				**1**	16	17
				**2**	20	20
				**3**		3
ccmFc	0.99	21	27			
				**1**	10	12
				**2**	11	11
				**3**		4
ccmFn	90.07	37	41			
				**1**	15	16
				**2**	19	22
				**3**	3	3
cob	4.69	15	16			
				**1**	9	9

				**2**	6	6
				**3**		1
cox1	13.37	6	6			
				**1**	3	3
				**2**	2	2
				**3**	1	1
cox2	8.08	14	16			
				**1**	4	5
				**2**	10	10
				**3**		1
cox3	11.08	13	15			
				**1**	3	3
				**2**	10	11
				**3**		1
*eleind009**	2.06	0	2			
				**2**		1
				**3**		1
matR	2.37	13	15			
				**1**	3	3
				**2**	10	10
				**3**		2
mttB	2.31	28	31			
				**1**	14	15
				**2**	14	15

				**3**		1
nad1	4.06	2	21			
				**1**		10
				**2**	2	8
				**3**		3
nad2	3.94	26	33			
				**1**	8	10
				**2**	18	19
				**3**		4
nad3	2.22	0	14			
				**1**		5
				**2**		9
nad4	4.09	19	22			
				**1**	5	7
				**2**	14	14
				**3**		1
nad4L	2.39	9	9			
				**1**	1	1
				**2**	8	8
nad5	5.34	10	13			
				**1**	2	2
				**2**	8	9
				**3**		2
nad6	5.29	11	12			

				**1**	3	4
				**2**	7	7
				**3**	1	1
nad7	5.01	25	26			
				**1**	7	7
				**2**	18	18
				**3**		1
nad9	5.58	15	15			
				**1**	4	4
				**2**	8	8
				**1**	3	3
rpl16	8.55	6	11			
				**2**	5	6
				**3**	1	5
rps12	3.97	6	6			
				**1**	2	2
				**2**	4	4
rps1	366.55	4	5			
				**2**	3	4
				**3**	1	1
rps13	0.89	5	5			
				**1**	2	2
				**2**	3	3
rps19	2.37	4	6			

				**1**	1	1
				**2**	3	3
				**3**		2
rps2	2.71	8	9			
				**1**	3	3
				**2**	5	5
				**3**		1
rps3	3.78	11	14			
				**1**	4	5
				**2**	7	8
				**3**		1
rps4	1.13	13	15			
				**1**	3	3
				**2**	10	10
				**3**		2
rps7	1.84	1	3			
				**1**		1
				**2**	1	1
				**3**		1
total codon 1					147	175
total codon 2					274	305
total codon 3					10	50
**total**					431	530

Supplementary Table 5. Number of differences among mitochondrial components of subunits within chloroids.

Subunit	Total sites	Mean no.diff.	Mean no. diff. per site
nad	8212	1.25	0.000153
mtt	752	1.33	0.001773
rpl	557	1.33	0.002394
cob	1163	2.67	0.002293
cox	3123	3.66	0.001173
mat	2036	4.67	0.002292
ccm	4409	4.69	0.001065
rps	4902	7.01	0.001431
atp	3375	11.94	0.003537
