# Supplementary material for: The Mitochondrial Genome of Eleusine indica and Characterization of Gene Content within Poaceae
Source: Genome Biol Evol. 2019 Oct 23;12(1):3684–97. doi: 10.1093/gbe/evz229 (PMC7145533; doi:10.1093/gbe/evz229)
Supplement: evz229_Supplementary_Data [file evz229_supplementary_data.zip › sup_figures_1-5_GBE_rev.pdf]

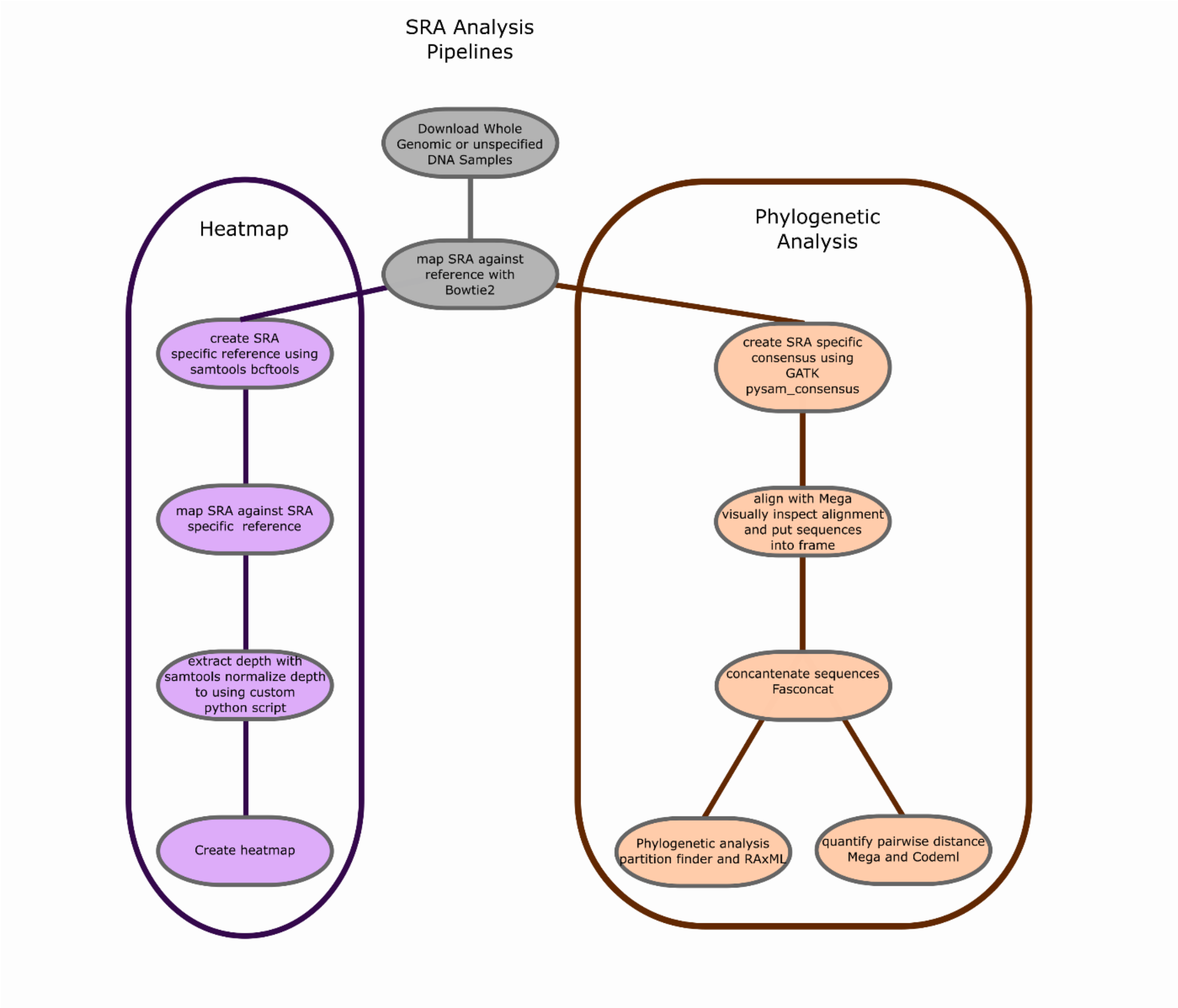

Supplementary Figure 1. Flowchart for handling SRA data

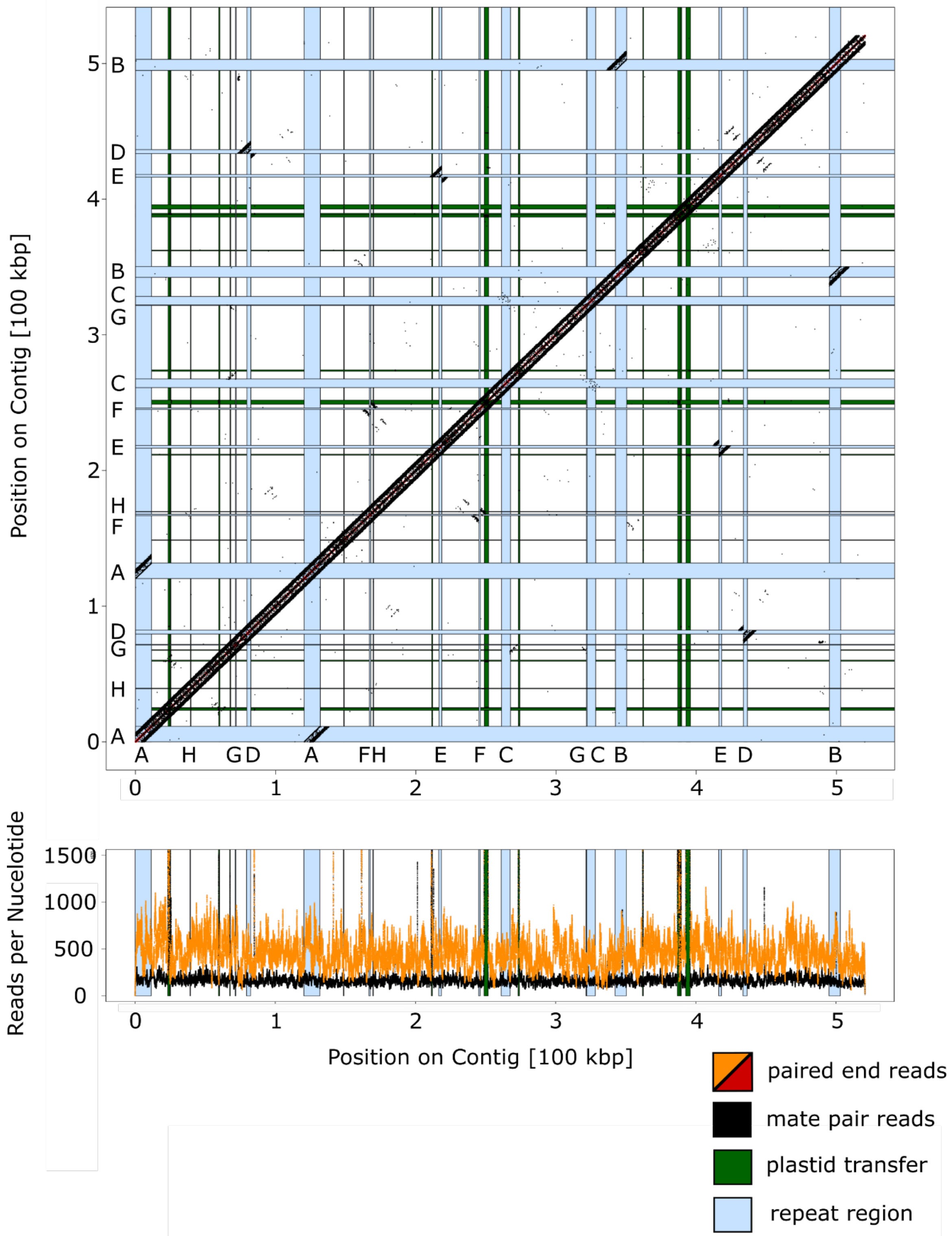

Position on Contig [100 kbp]
Reads per Nucelotide
Position on Contig [100 kbp]
A H GD A FH E F C GC B E D B
A H G D F H E F C G C B E D B
paired end reads
mate pair reads
plastid transfer
repeat region

Supplementary Figure 2 a) Plots the relationship of a read to its mate in black or to its pair in red. If the assembly is discordant, a white "X" shaped gap is produced along the x=y line because of the absence of reads spanning that junction. These gaps are expected to occur at or around the borders of repeat regions indicated in blue or the borders of plastid regions indicated in green. The absence of these gaps supports concordant assembly, they do not rule out the existence of alternate, potentially under sampled conformations arising within our sequenced reads. b) Depth of concordant mapping reads in Bowtie 2 for both paired-end and mate-pair reads. Mitochondrial coverage is around 400 reads/bp for paired-end (Supplementary Table 1). Green regions indicate plastid inserts and coverage spikes from plastid reads matching these regions.

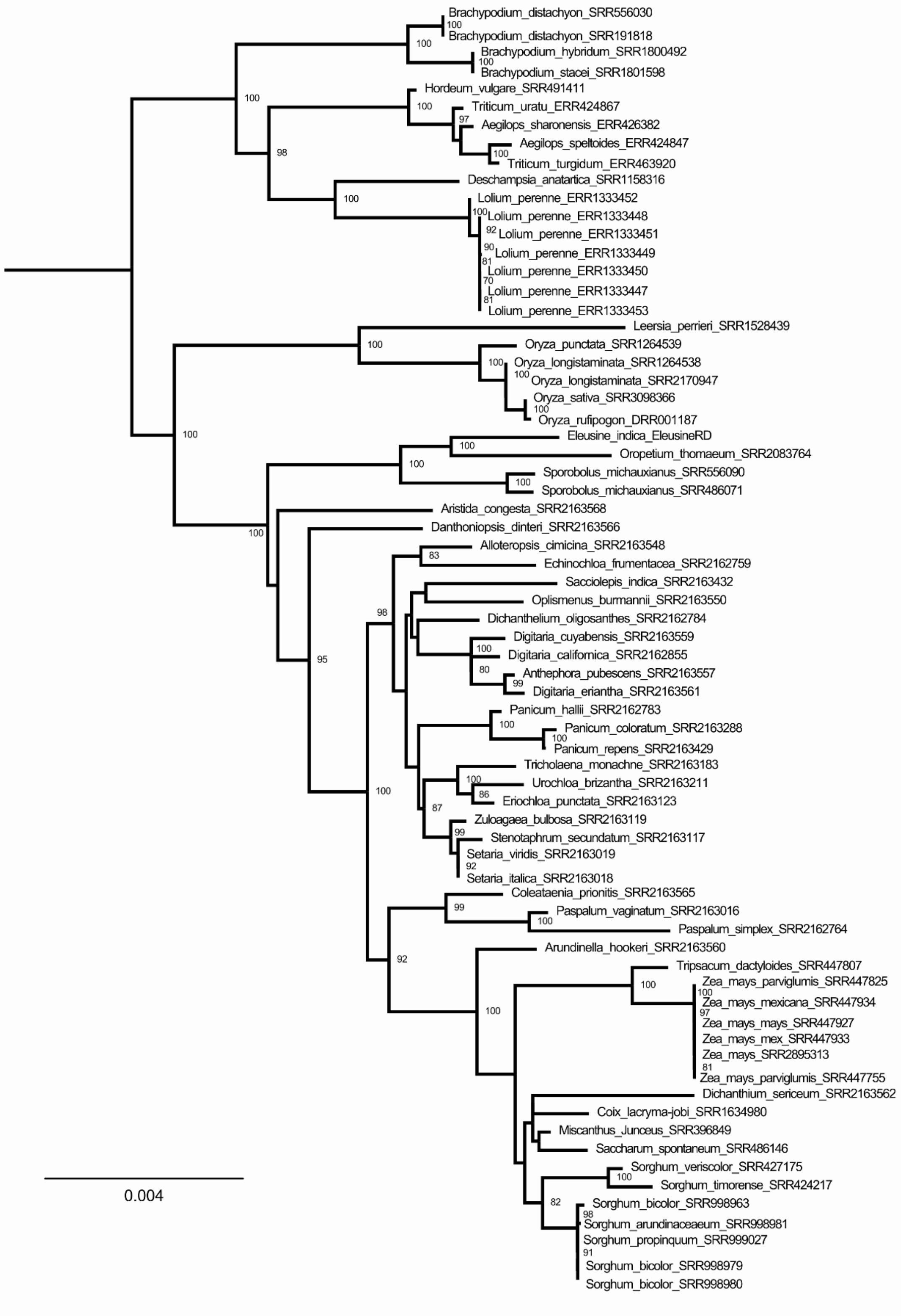
Brachypodium_distachyon_SRR556030
Brachypodium_distachyon_SRR191818
Brachypodium_hybridum_SRR1800492
Brachypodium_stacei_SRR1801598
Hordeum_vulgare_SRR491411
Triticum_uratu_ERR424867
Aegilops_sharonensis_ERR426382
Aegilops_speltoides_ERR424847
Triticum_turgidum_ERR463920
Deschampsia_anatartica_SRR1158316
Lolium_perenne_ERR1333452
Lolium_perenne_ERR1333448
Lolium_perenne_ERR1333451
Lolium_perenne_ERR1333449
Lolium_perenne_ERR1333450
Lolium_perenne_ERR1333447
Lolium_perenne_ERR1333453
Leersia_perrieri_SRR1528439
Oryza_punctata_SRR1264539
Oryza_longistaminata_SRR1264538
Oryza_longistaminata_SRR2170947
Oryza_sativa_SRR3098366
Oryza_rufipogon_DRR001187
Eleusine_indica_EleusineRD
Oropetium_thomaeum_SRR2083764
Sporobolus_michauxianus_SRR556090
Sporobolus_michauxianus_SRR486071
Aristida_congesta_SRR2163568
Danthoniopsis_dinteri_SRR2163566
Alloteropsis_cimicina_SRR2163548
Echinochloa_frumentacea_SRR2162759
Sacciolepis_indica_SRR2163432
Oplismenus_burmannii_SRR2163550
Dichanthelium_oligosanthes_SRR2162784
Digitaria_cuyabensis_SRR2163559
Digitaria_californica_SRR2162855
Anthephora_pubescens_SRR2163557
Digitaria_eriantha_SRR2163561
Panicum_hallii_SRR2162783
Panicum_coloratum_SRR2163288
Panicum_repens_SRR2163429
Tricholaena_monachne_SRR2163183
Urochloa_brizantha_SRR2163211
Eriochloa_punctata_SRR2163123
Zuloagaea_bulbosa_SRR2163119
Stenotaphrum_secundatum_SRR2163117
Setaria_viridis_SRR2163019
Setaria_italica_SRR2163018
Coleataenia_prionitis_SRR2163565
Paspalum_vaginatum_SRR2163016
Paspalum_simplex_SRR2162764
Arundinella_hookeri_SRR2163560
Tripsacum_dactyloides_SRR447807
Zea_mays_parviglumis_SRR447825
Zea_mays_mexicana_SRR447934
Zea_mays_mays_SRR447927
Zea_mays_mex_SRR447933
Zea_mays_SRR2895313
Zea_mays_parviglumis_SRR447755
Dichanthium_sericeum_SRR2163562
Coix_lacryma-jobi_SRR1634980
Miscanthus_Junceus_SRR396849
Saccharum_spontaneum_SRR486146
Sorghum_veriscolor_SRR427175
Sorghum_timorense_SRR424217
Sorghum_bicolor_SRR998963
Sorghum_arundinaceaeum_SRR998981
Sorghum_propinquum_SRR999027
Sorghum_bicolor_SRR998979
Sorghum_bicolor_SRR998980
0.004

Supplementary Figure 3

Maximum likelihood tree inferred from consensus mt sequences using codon by gene partitioning scheme. Partitions identified in partitionfinder, RAxML analysis with GTRGAMMA for 100 bootstraps. All OTUs had at least 10,000 bp of sequence. ML tree created from concatenated alignment of *atp1*, *atp4*, *atp6*, *atp8*, *atp9*, *ccmB*, *ccmC*, *ccmFC_e1*, *ccmFC_e2*, *ccmFN*, *cob*, *cox1*, *cox2_e1*, *cox2_e2*, *cox3*, *matR*, *mttB*, *nad1_et1*, *nad1_et3*, *nad1_et5*, *nad2_et1*, *nad2_et2*, *nad2_et3*, *nad2_et4*, *nad2_et5*, *nad3*, *nad4L*, *nad4_e1*, *nad4_e2*, *nad4_e3*, *nad4_e4*, *nad5_e3*, *nad5_et1*, *nad5_et2*, *nad5_et4*, *nad5_et5*, *nad6*, *nad7_e1*, *nad7_e3*, *nad7_e4*, *nad7_e5*, *nad9*, *rpl16*, *rps12*, *rps13*, *rps19*, *rps1*, *rps3_e1*, *rps4* and *rps7*. Bootstrap values of 80 or greater are shown. Tree rooted on *Ananas comosus* (GenBank accession DRR022930) not shown.

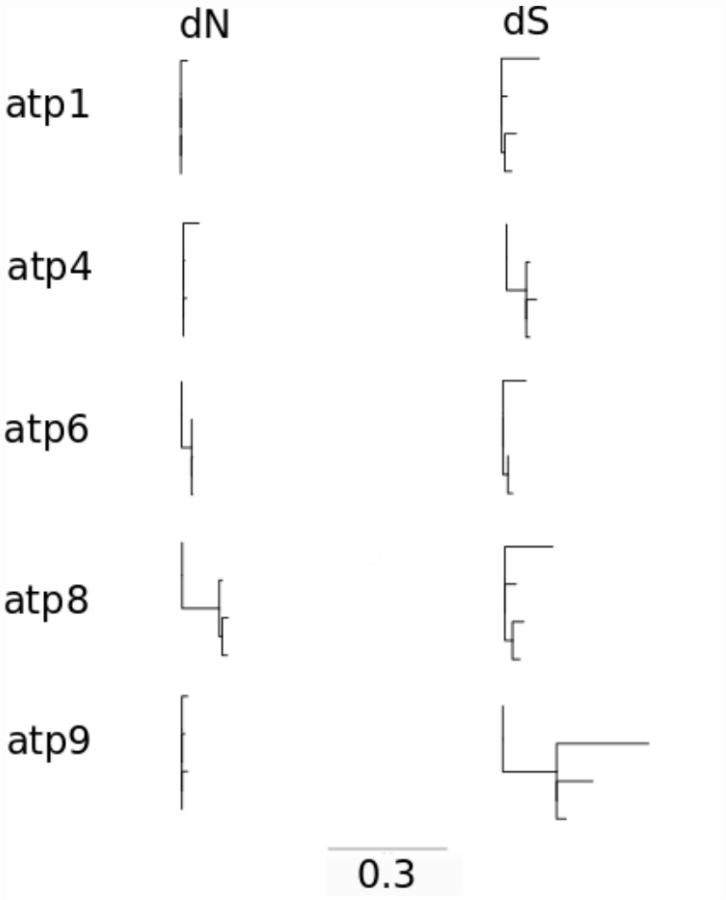

Supplementary Figure 4 Tree lengths taken from codeml comparing the rate of synonymous to non-synonymous substitutions for mt genes encoding atpase subunits.

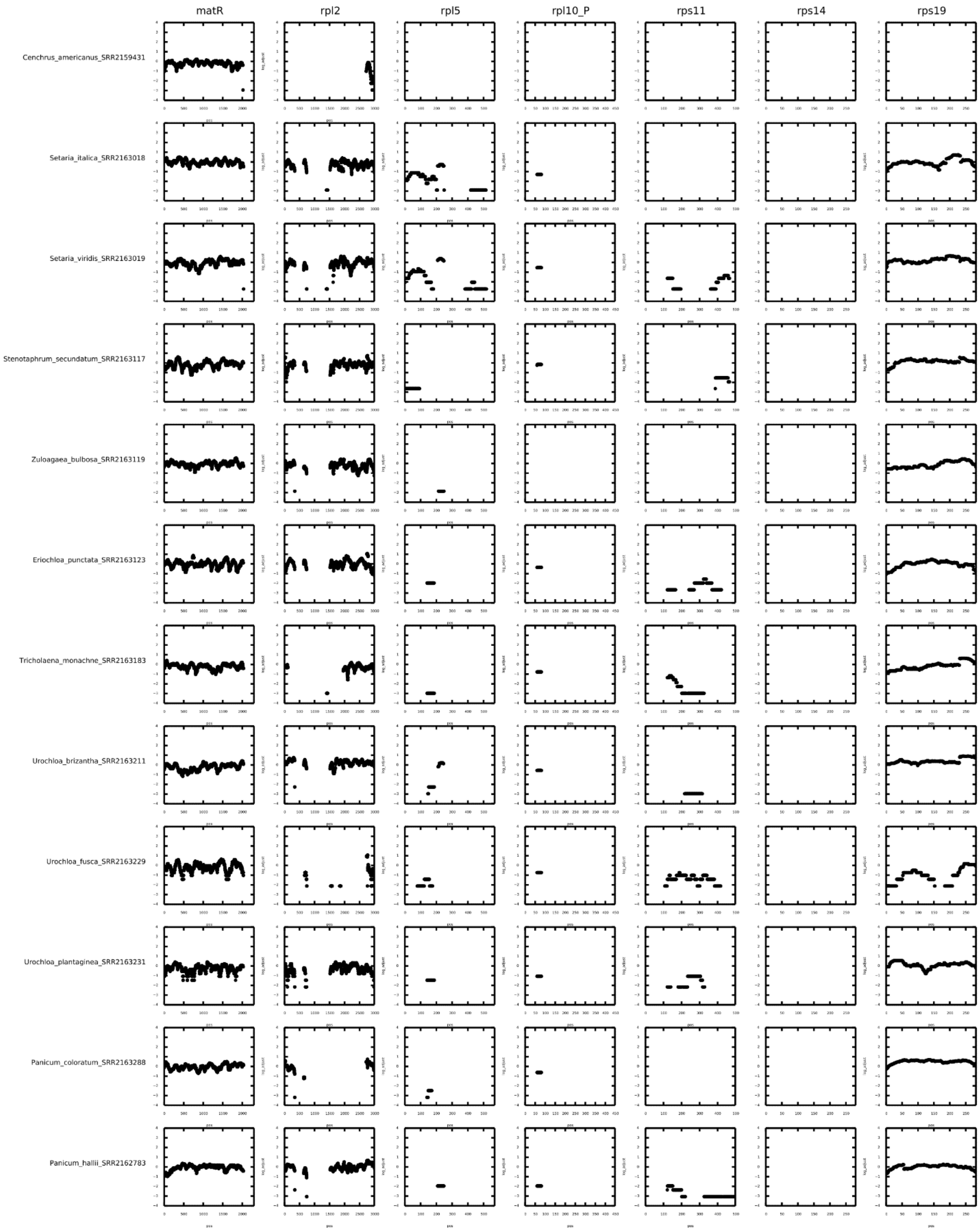

matR
rpl2
rpl5
rpl10_P
rps11
rps14
rps19
Cenchrus_americanus_SRR2159431
Setaria_italica_SRR2163018
Setaria_viridis_SRR2163019
Stenotaphrum_secundatum_SRR2163117
Zuloagaea_bulbosa_SRR2163119
Eriochloa_punctata_SRR2163123
Tricholaena_monachne_SRR2163183
Urochloa_brizantha_SRR2163211
Urochloa_fusca_SRR2163229
Urochloa_plantaginea_SRR2163231
Panicum_coloratum_SRR2163288
Panicum_hallii_SRR2162783

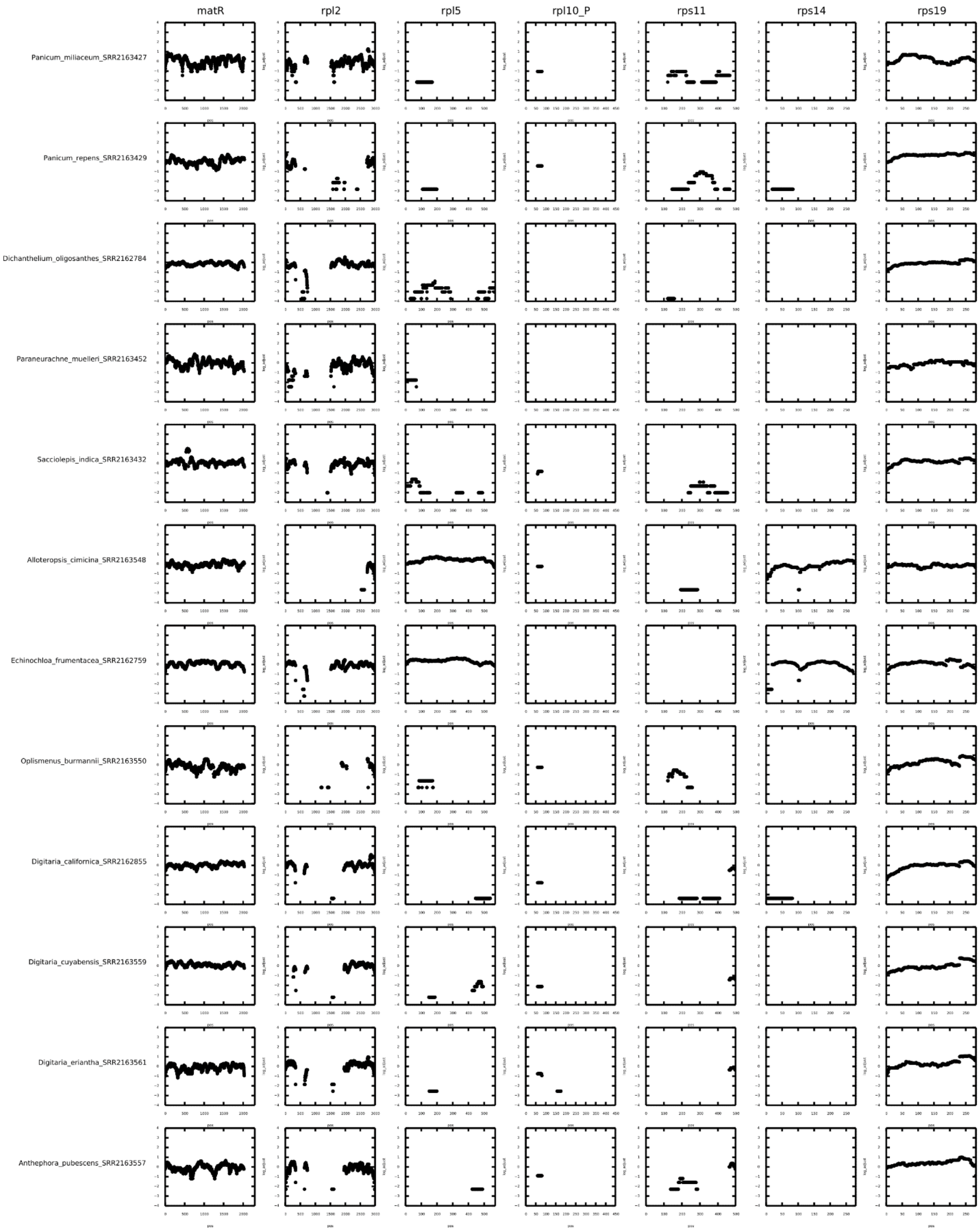

matR
rpl2
rpl5
rpl10_P
rps11
rps14
rps19
Panicum_miliaceum_SRR2163427
Panicum_repens_SRR2163429
Dichanthelium_oligosanthes_SRR2162784
Paraneurachne_muelleri_SRR2163452
Sacciolepis_indica_SRR2163432
Alloteropsis_cimicina_SRR2163548
Echinochloa_frumentacea_SRR2162759
Oplismenus_burmannii_SRR2163550
Digitaria_californica_SRR2162855
Digitaria_cuyabensis_SRR2163559
Digitaria_eriantha_SRR2163561
Anthephora_pubescens_SRR2163557

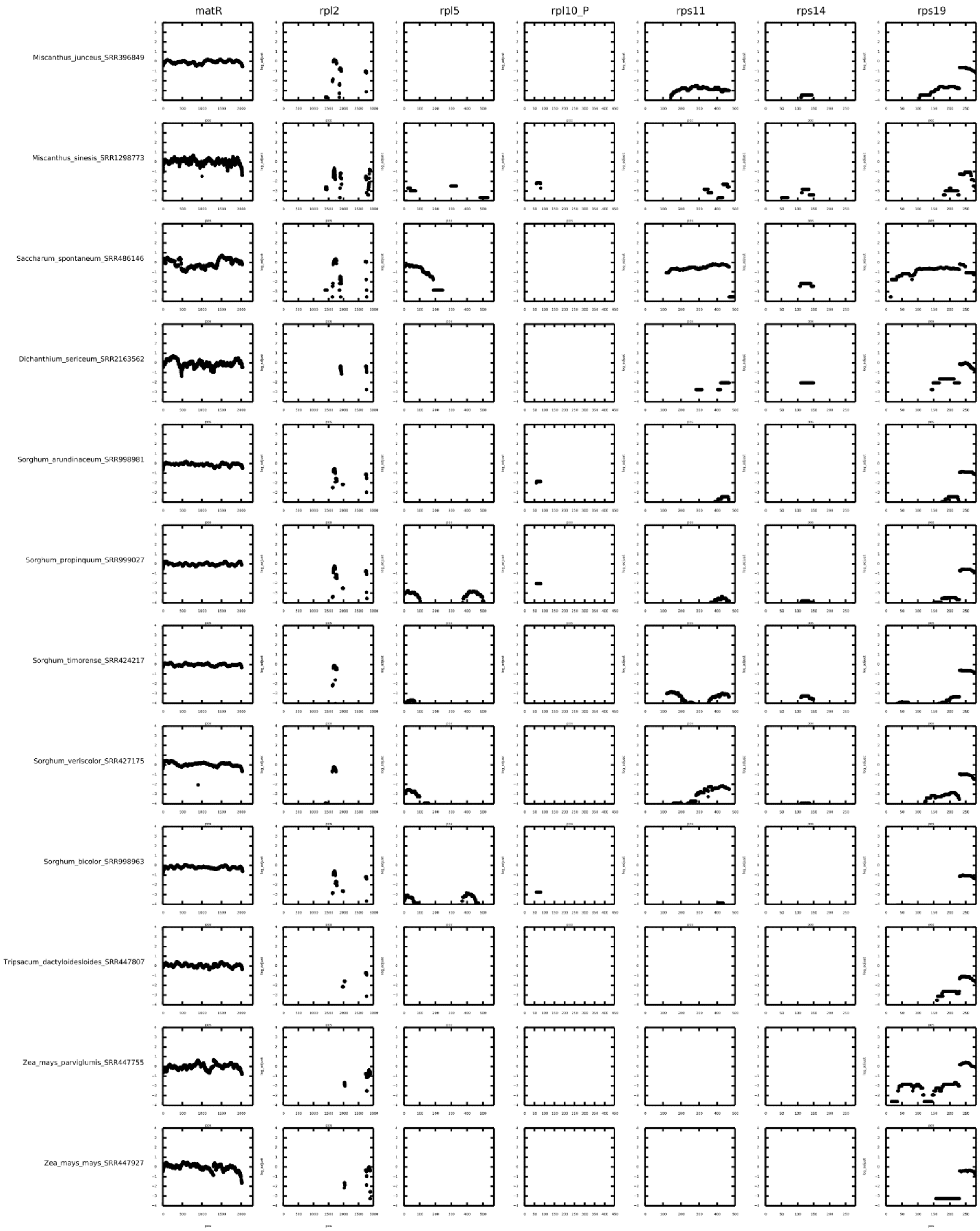
matR
rpl2
rpl5
rpl10_P
rps11
rps14
rps19
Miscanthus_junceus_SRR396849
Miscanthus_sinesis_SRR1298773
Saccharum_spontaneum_SRR486146
Dichanthium_sericeum_SRR2163562
Sorghum_arundinaceum_SRR998981
Sorghum_propinquum_SRR999027
Sorghum_timorense_SRR424217
Sorghum_veriscolor_SRR427175
Sorghum_bicolor_SRR998963
Tripsacum_dactyloidesloides_SRR447807
Zea_mays_parviglumis_SRR447755
Zea_mays_mays_SRR447927

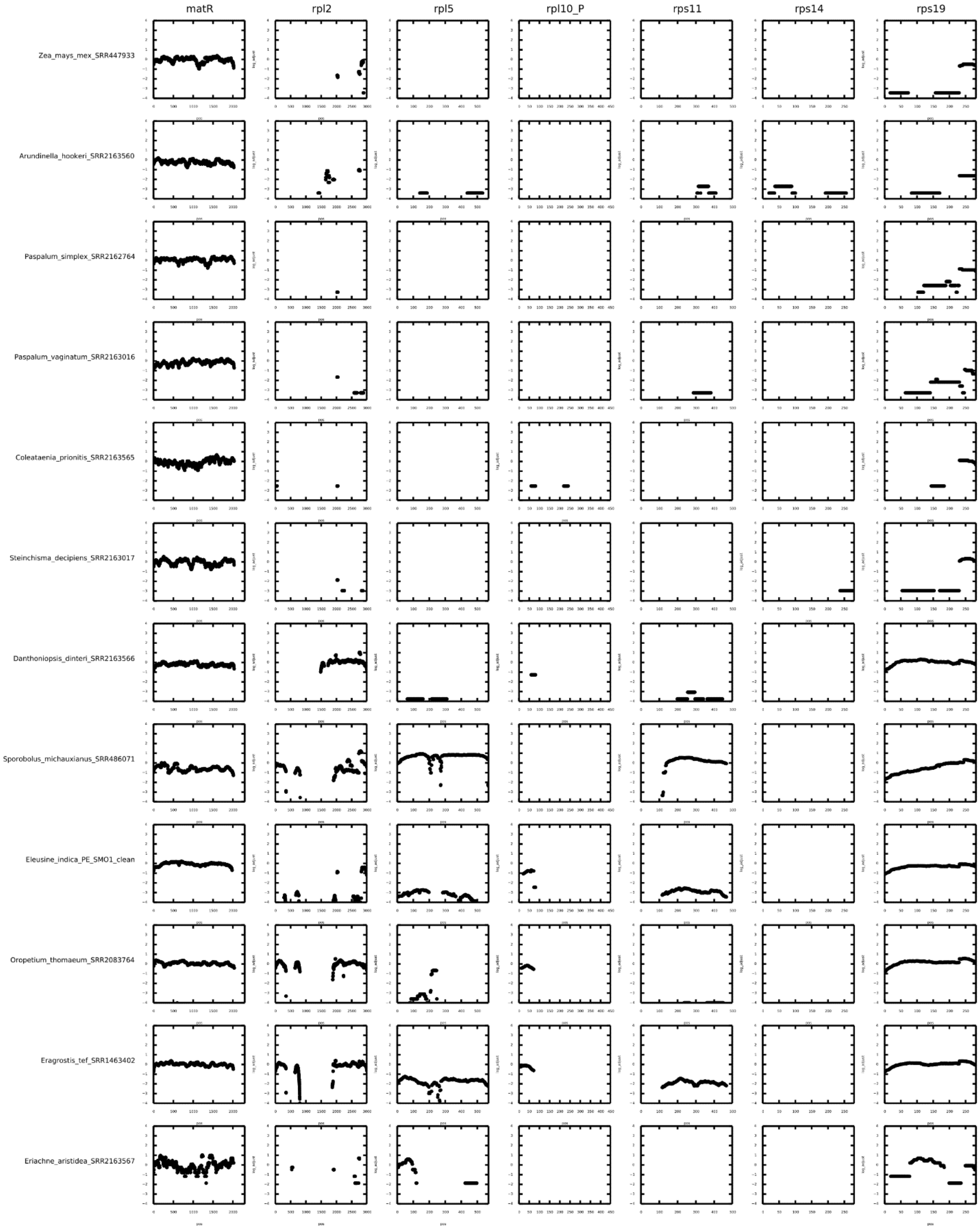

matR
rpl2
rpl5
rpl10_P
rps11
rps14
rps19
Zea_mays_mex_SRR447933
Arundinella_hookeri_SRR2163560
Paspalum_simplex_SRR2162764
Paspalum_vaginatum_SRR2163016
Coleataenia_prionitis_SRR2163565
Steinchisma_decipiens_SRR2163017
Danthoniopsis_dinteri_SRR2163566
Sporobolus_michauxianus_SRR486071
Eleusine_indica_PE_SMO1_clean
Oropetium_thomaeum_SRR2083764
Eragrostis_tef_SRR1463402
Eriachne_aristidea_SRR2163567

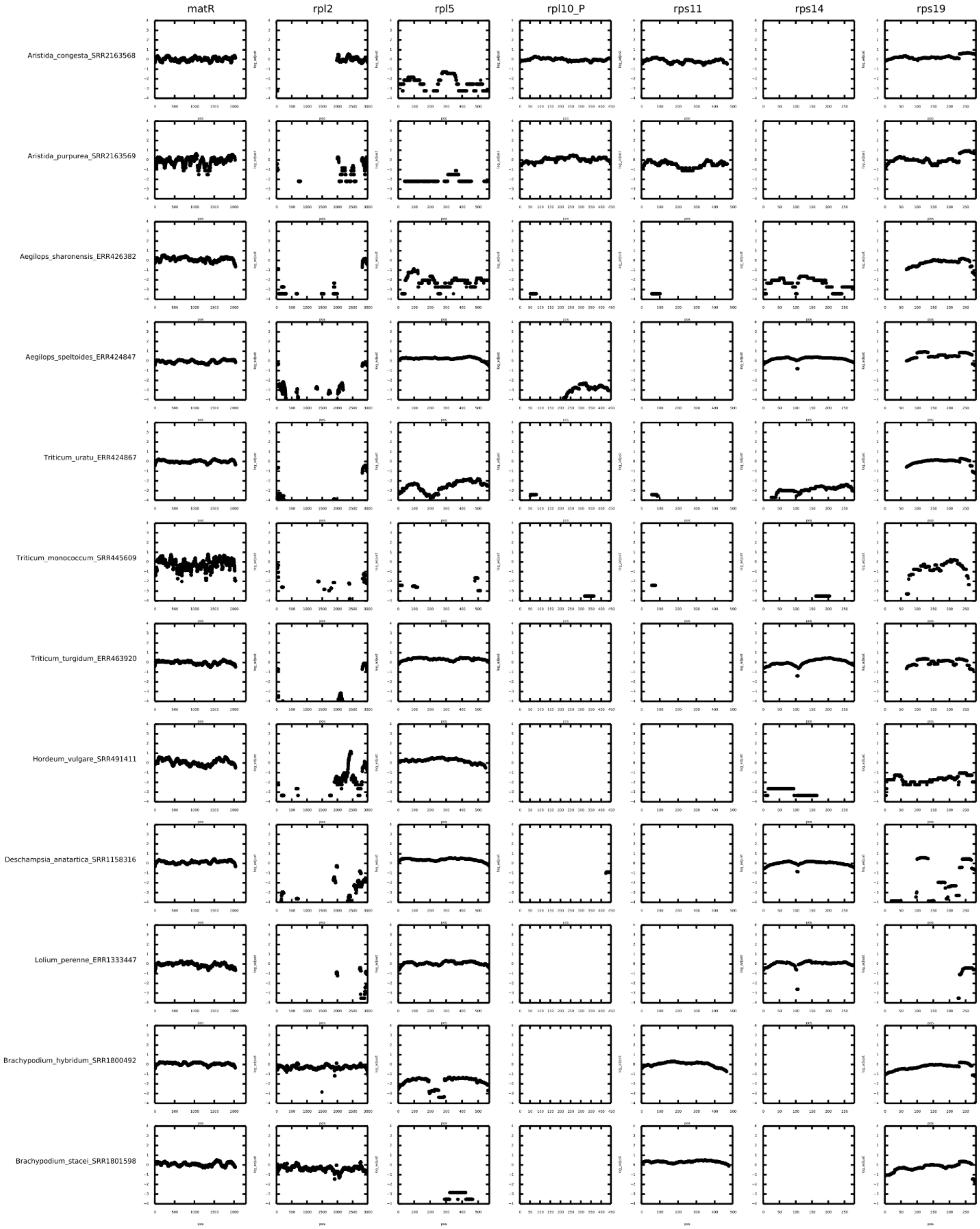
matR
rpl2
rpl5
rpl10_P
rps11
rps14
rps19
Aristida_congesta_SRR2163568
Aristida_purpurea_SRR2163569
Aegilops_sharonensis_ERR426382
Aegilops_speltoides_ERR424847
Triticum_uratu_ERR424867
Triticum_monococcum_SRR445609
Triticum_turgidum_ERR463920
Hordeum_vulgare_SRR491411
Deschampsia_anatartica_SRR1158316
Lolium_perenne_ERR1333447
Brachypodium_hybridum_SRR1800492
Brachypodium_stacei_SRR1801598

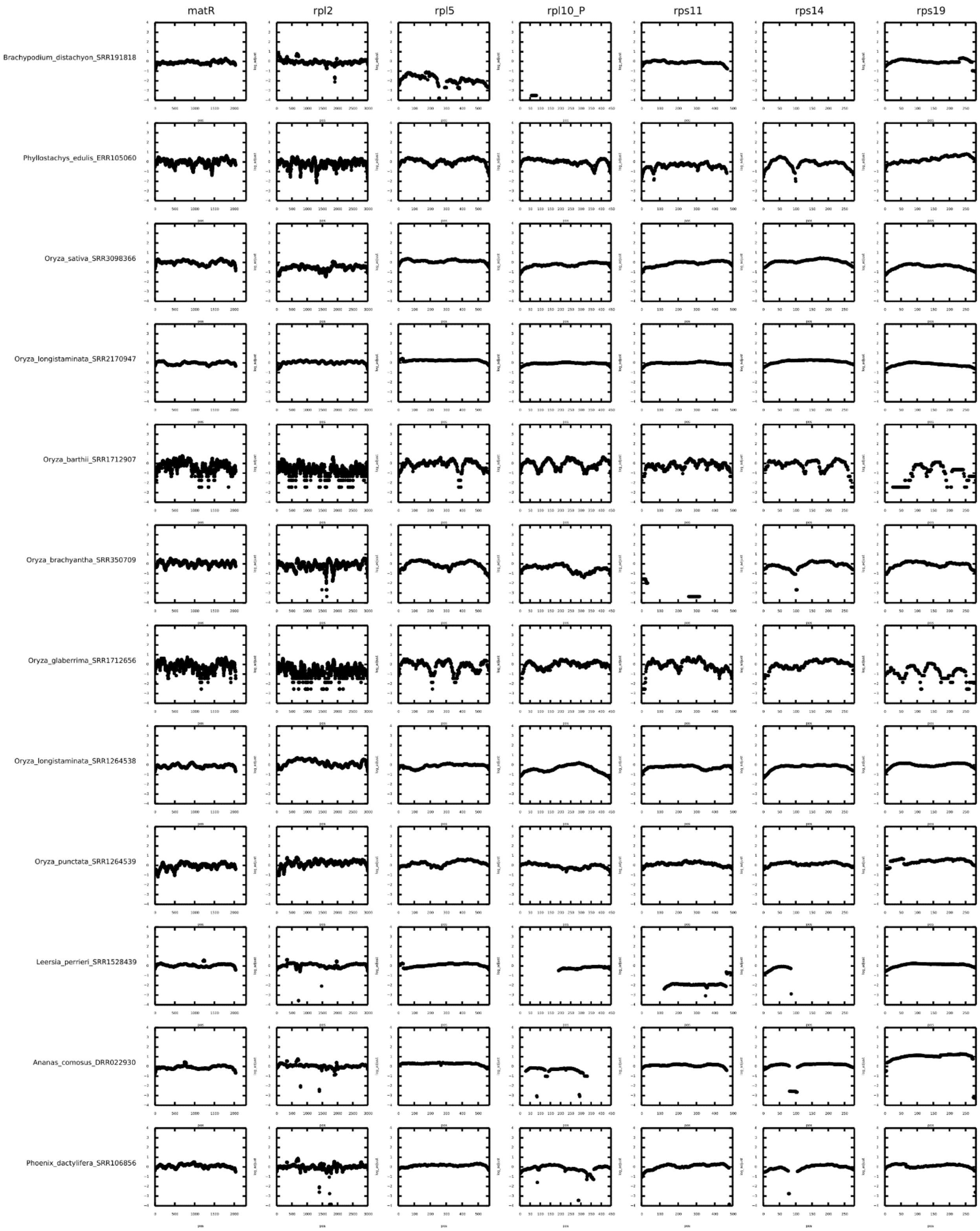

Supplementary Figure 5 Natural log of the mt fold depth plus 1e-10 of mapping for each read set across selected genes known to be absent. The gene *matR* is used as a positive control.
